# Role of mucus-bacteria interactions in Enterotoxigenic *Escherichia coli* (ETEC) H10407 virulence and interplay with human microbiome

**DOI:** 10.1038/s41522-022-00344-6

**Published:** 2022-10-20

**Authors:** Thomas Sauvaitre, Josefien Van Landuyt, Claude Durif, Charlène Roussel, Adeline Sivignon, Sandrine Chalancon, Ophélie Uriot, Florence Van Herreweghen, Tom Van de Wiele, Lucie Etienne-Mesmin, Stéphanie Blanquet-Diot

**Affiliations:** 1 Université Clermont Auvergne, INRAE, UMR 454 MEDIS, Microbiologie Environnement Digestif et Santé (MEDIS), CRNH Auvergne, 63000 Clermont-Ferrand, France; 2 Ghent University, Faculty of Bioscience Engineering, Center for Microbial Ecology and Technology (CMET), Ghent, Belgium; 3 Université Laval, Nutrition and Functional Foods Institute (INAF), 2440 Bd Hochelaga Suite 1710, Québec, QC G1V 0A6 Canada; 4 Université Clermont Auvergne, UMR 1071 Inserm, USC-INRAE 2018, Microbes, Intestin, Inflammation et Susceptibilité de l’Hôte (M2iSH), 63000 Clermont-Ferrand, France

**Keywords:** Pathogens, Biofilms, Microbiome

## Abstract

The intestinal mucus layer has a dual role in human health constituting a well-known microbial niche that supports gut microbiota maintenance but also acting as a physical barrier against enteric pathogens. Enterotoxigenic *Escherichia coli* (ETEC), the major agent responsible for traveler’s diarrhea, is able to bind and degrade intestinal mucins, representing an important but understudied virulent trait of the pathogen. Using a set of complementary in vitro approaches simulating the human digestive environment, this study aimed to describe how the mucus microenvironment could shape different aspects of the human ETEC strain H10407 pathophysiology, namely its survival, adhesion, virulence gene expression, interleukin-8 induction and interactions with human fecal microbiota. Using the TNO gastrointestinal model (TIM-1) simulating the physicochemical conditions of the human upper gastrointestinal (GI) tract, we reported that mucus secretion and physical surface sustained ETEC survival, probably by helping it to face GI stresses. When integrating the host part in Caco2/HT29-MTX co-culture model, we demonstrated that mucus secreting-cells favored ETEC adhesion and virulence gene expression, but did not impede ETEC Interleukin-8 (IL-8) induction. Furthermore, we proved that mucosal surface did not favor ETEC colonization in a complex gut microbial background simulated in batch fecal experiments. However, the mucus-specific microbiota was widely modified upon the ETEC challenge suggesting its role in the pathogen infectious cycle. Using multi-targeted in vitro approaches, this study supports the major role played by mucus in ETEC pathophysiology, opening avenues in the design of new treatment strategies.

## Introduction

Continuously produced and secreted by goblet cells, the intestinal mucus is a complex viscoelastic adherent secretion. The mucus is composed of water, electrolytes, lipids and large glycoproteins called mucins^1,2^. Due to its location at the interface between the digestive lumen and the host, accumulating evidence has shown the mucus layer to be a key feature in the modulation of gut health, mainly through the modulation of the gut microbiome^3,4^. From one side, the mucus layer is well-known to be a microbial niche with particular environmental conditions (e.g. concentrated immune molecules and higher oxygen concentration) and nutrient sources, allowing its colonisation by a specific microbiota, with higher abundance of Firmicutes, Proteobacteria and Actinobacteria species^5^. Mucosal communities are highly enriched in the *Bacteroides acidifaciens*, *Bacteroides fragilis*, the mucin-degrader *Akkermansia muciniphila* and in species belonging to the *Clostridia* class^6–8^. On the other side, mucus acts as a barrier against physical, chemical and biological stressors^2,9^. Notably, enteric pathogens have to interact with and penetrate this line of defense in order to colonize the intestinal epithelium^10–12^. Several studies have shown that genetic or environmental defects in mucus integrity increase pathogen susceptibility^10,13^. Among enteric pathogens, enterotoxigenic *Escherichia coli* (ETEC) is known to possess virulence factors to interact with and penetrate the mucus layer^14,15^. The food and water-borne ETEC is one of the most important cause of travelers’ diarrhea, with hundreds of millions of diarrheal episodes worldwide^16^. Once ingested, ETEC first has to withstand the stringent conditions (e.g. acidic pH, bile acids and competition with gut microbes) encountered in the human digestive environment^17,18^, to reach its action site in the distal part of the small intestine^19–21^. Then, the two characterized ETEC mucin-degrading proteins (namely mucinases), EatA and YghJ, promote access to the underlying epithelium^14,15^ and a panel of fimbrial (e.g. FimH) and non-fimbrial (e.g. Tia) adhesins supports bacterial attachment to the mucosal surface through the recognition of specific surface receptors^22–24^. Some of these surface receptors have patterns specific to mucus. As an example, the EtpA adhesin preferentially binds to N-acetylgalactosamine motifs, which are expressed in blood group A antigens^25–27^. The mucolytic action of ETEC layer and adhesion to the epthelial surface facilitate the production and delivery of heat labile (LT) and/or heat-stable (ST) enterotoxins. Such toxins play a major role in ETEC pathogenesis, leading to profuse watery diarrhea^28^. In turn, the LT toxin also alters the structure and composition of the intestinal epithelial mucin layer by decreasing MUC4 expression^29^ and increasing MUC2 expression and secretion, which results in an increased pathogen adhesion^30,31^. ETEC also induces an intestinal inflammatory response (notably an IL-8 secretion) that correlates with disease severity^32–34^. Such phenomenon can be in turn modulated by ETEC virulence factors, as LT/ST toxins and the YghJ mucinase^35–38^. Lastly, several human clinical trials and in vitro reports have shown that ETEC infection is associated with alterations of gut microbiota in terms of structure and activity^18,39–42^, suggesting its possible involvement in host susceptibility to the pathogen^43^.

Given the modulatory role between mucus and enteric pathogen virulence and the scarcity of data regarding ETEC pathotype, the present study aims to decipher more closely the role of bacteria-mucus interactions in ETEC infection. Using complementary in vitro approaches simulating the human gastrointestinal tract, we investigated the role of the mucus layer on various facets of ETEC reference strain H10407 physiopathology, namely survival, adhesion, IL-8 induction, virulence and interactions with fecal microbiota.

## Results

### ETEC was able to grow on mucin as sole substrate

ETEC strain H10407 ability to use purified mucin as substrate was first assayed following pathogen growth kinetics in M9 minimal culture medium supplemented with or without commercially available mucins (Type II and III) (Supplementary Fig. 1). After a 5-hour incubation period, the number of cultivable ETEC cells was multiplied by 56 and 32 compared to the control condition, with commercial type II and type III mucins, respectively (*p* < 0.05). The capacity of ETEC to grow on type II mucin was significantly higher compared to type III, with 6.6 × 10^8^ versus 3.9 × 10^8^ CFU.mL^−1^ (Colony Forming Units per mL) at the end of the experiment (*p* < 0.05, *n* = 3, Tukey’s multiple comparison tests).

### ETEC showed a tropism for mucin and mucus-secreting intestinal cells

**Fig. 1:**
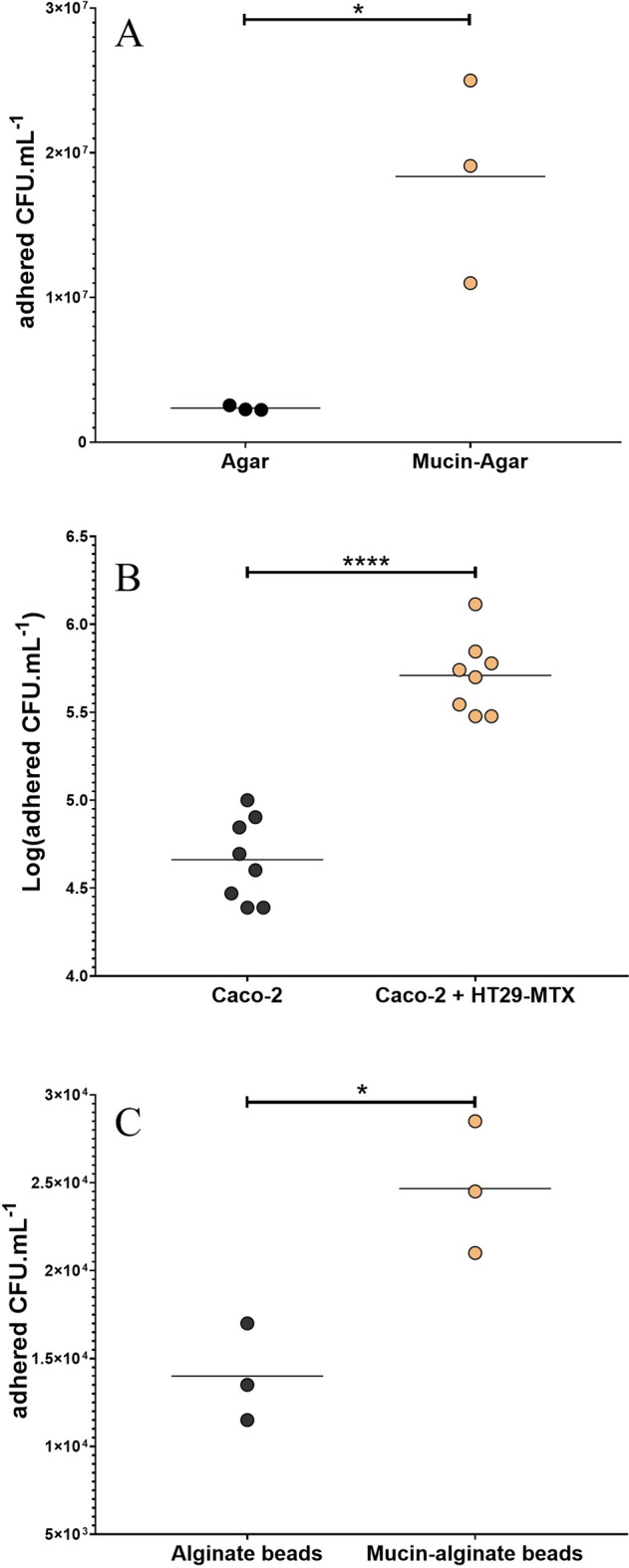
ETEC adhesion to the mucus compartment. Adhesion of the ETEC strain H10407 to the mucus compartment was analyzed by three different in vitro assays. **A** ETEC bacteria (initial concentration: 10^7^ CFU.ml^−1^) adhered in plate assays to type II mucin-agar layer (orange dots) or agar without mucin used as a negative control (black dots) after one hour exposure. **B** ETEC adhesion to Caco-2/HT29-MTX co-culture model (orange dots) or Caco-2 cells only (black dots) after infection at MOI 100 for 3 h. **C** ETEC bacteria (initial concentration: 10^7^ CFU.ml^−1^) adhered to type III mucin-alginate beads (orange dots) or alginate without mucin used as a negative control (black dots), after one hour and half static gastro-ileal digestion procedure. Figures represent the results of three independent experiments (in **B**, all technical replicates are represented). Means are indicated by black bars. *p*-values are provided by unpaired *t* test with Welch’s correction (*p < 0.05; ****p < 0.0001).

Specificity of ETEC adhesion to mucus was evaluated using different in vitro models (Fig. 1). In a simple plate assay, ETEC strain H10407 showed an enhanced adhesion for mucin-agar layer (with type II mucin) compared to agar alone (Fig. 1A), with an average of 1.8 × 10^7^ *versus* 2.4 × 10^6^ CFU.mL^−1^ adhered bacteria (*p* < 0.05), respectively. The host component was then integrated by performing cell adhesion experiments, including a monoculture of Caco-2 (enterocytes) or a co-culture of Caco-2/HT29-MTX (enterocyte and mucus-secreting goblet cells) (Fig. 1B). After 3-hour adhesion test, the number of adherent ETEC was half one log higher in the co-culture of Caco-2/HT29-MTX cells compared to Caco-2 alone (5.75 *versus* 5.23 logCFU.mL^−1^), suggesting a tissue tropism of ETEC towards the mucus-secreting cells (*p* < 0.0001).

### Mucin allowed ETEC to better cope with upper gastrointestinal stresses

**Fig. 2:**
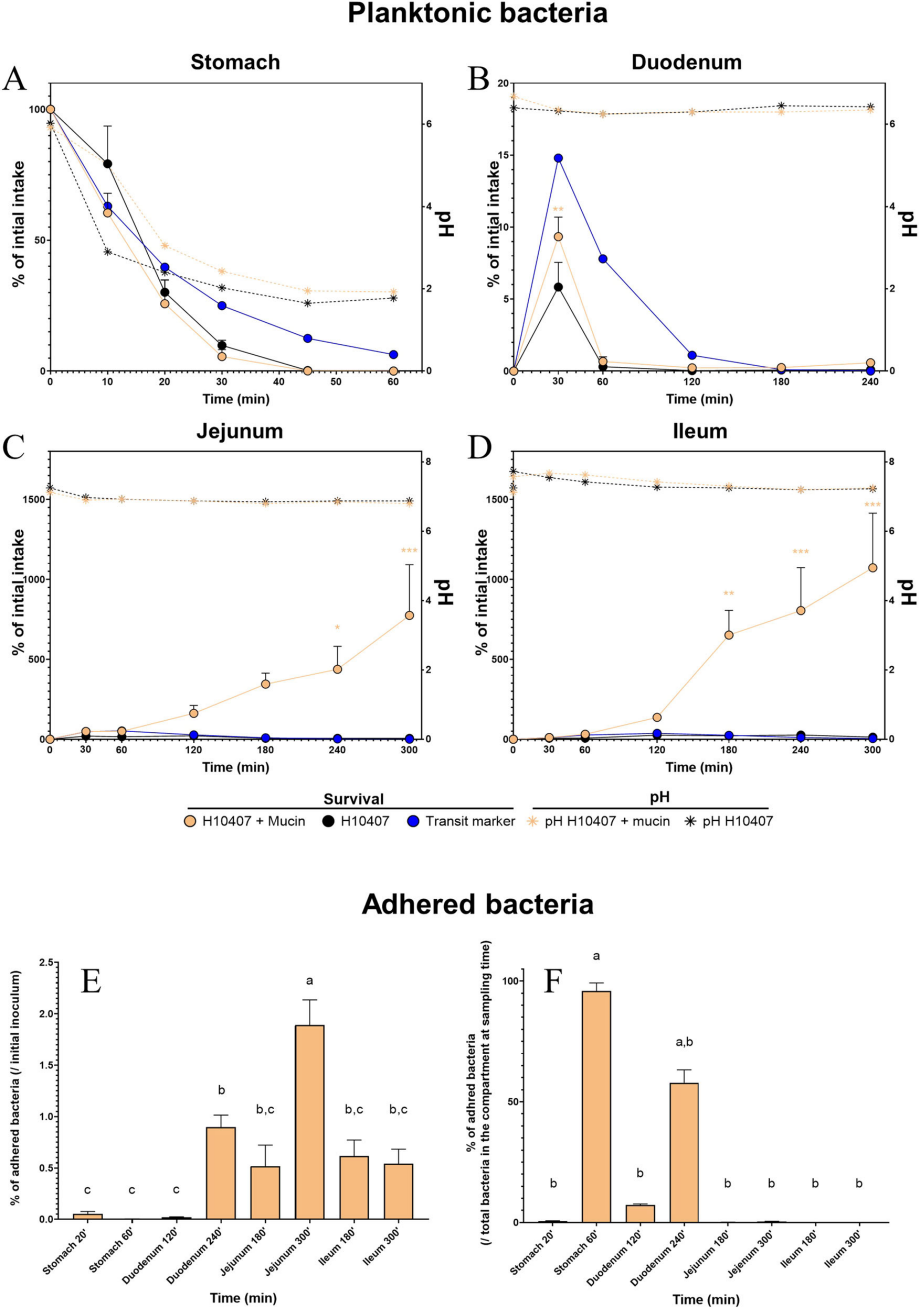
Dynamics of ETEC survival and adhesion to mucin in the successive TIM-1 compartments. **A–D** After introduction of a glass of ETEC-contaminated water (10^10^ CFU) in the TIM-1 model, the number of ETEC bacteria in the lumen (“planktonic” bacteria) of stomach (**A**), duodenum (**B**), jejunum (**C**) and ileum (**D**) compartments was determined by plate counting. Results are expressed as mean percentages ± SEM (*n* = 4) of initial intake. Bacterial survival kinetics with (orange dots) and without (black dots) mucin are compared with an inert and non-absorbable transit marker indicating 100% survival (blue dots). The evolution of pH in each compartment is also indicated with (orange star) or without mucin (black star). Indicated *p* value correspond to times at which the survival in the mucin condition was found to be statistically different from the non-mucin condition according to Sidak multiple comparison tests (*p < 0.05; **p < 0.01; ***p < 0.001). **E, F** ETEC adhesion to mucin-alginate beads was also analyzed by sampling and plating at different time points in each compartment of TIM-1. Results are expressed as mean percentages ± SEM (*n* = 4) of initial intake (**E**) or of total bacteria (planktonic + adhered) in the compartment (**F**). Results that are not significantly different from each other according to Tukey’s multicomparison test are grouped under the same letter (*p* < 0.05).

To evaluate the impact of physicochemical parameters (pH, digestive secretions) of the upper human gastrointestinal tract on ETEC adhesion specificity to mucin, we first performed bead adhesion assays through a simple static in vitro digestion process (Fig. 1C, Table 1). After 180 min of digestion, ETEC strain H10407 showed a 1.8-fold higher adhesion on type III mucin-containing alginate beads compared to the control condition with alginate beads (*p* < 0.05). Then, the dynamic and multi-compartmental TIM-1 model was used to simulate more closely human physiological digestive conditions (Fig. 2). In this model, we assessed the effect of mucin secretion mimicking mucin shedding on ETEC survival in the digestive lumen but also its ability to adhere to mucin-alginate beads as a physical surface during transit. In the TIM-1 gastric compartment (Fig. 2A), mucin addition did not significantly modify planktonic bacteria survival resulting in significant mortality after 20 min of digestion, independent of the tested condition. In the duodenum of TIM-1 (Fig. 2B), mucin addition attenuated the observed ETEC decrease in viability, certainly due to stringent conditions (e.g. bile acids). Such phenomenon was significant at T30 min with survival percentage of 9.3% of the initial intake with mucin compared to 5.8% in the control condition (*p* < 0.01). At the end of the duodenal digestion (T240 min), ETEC began to multiply. Mucin reinforced this multiplication with 0.58% of bacterial intake viable compared to 0.10% in the non-mucin condition and 0.01 % for the transit marker (Fig. 2B). In the jejunal and ileal compartments (Fig. 2C, D), mucin addition elicited a remarkable and highly significant multiplication of ETEC, especially from T120 min of digestion. The ultimate time points (T240 and T300 min) are significantly different between the mucin and non-mucin conditions according to Sidak multiple comparison test (*p* < 0.05). ETEC global survival in the ileal effluents reached 28.9- and 0.6-fold of the initial intake under the mucin and control conditions, respectively (data not shown). The number of adherent bacteria on mucin-alginate beads was also determined across dynamic in vitro digestions. If the highest numbers of adherent bacteria were found in the distal small intestinal compartments, reaching nearly 2% of the initial bacterial intake at T300 min in the jejunum (Fig. 2E), the highest adhesion ratios (representing the percentage of adhered bacteria in each compartment) were observed in the stomach and duodenum of TIM-1 where harsh conditions are found (e.g. acidic pH and high bile salts concentrations). The percentage of adhered bacteria reached 90% in the stomach at 60 min (when pH reached 1.9) and 60% in the duodenum at 240 min (Fig. 2F).

**Table 1:** Static in vitro gastro-ileal digestion procedure.

Parameters of static in vitro digestion	Gastric vessel	Duodenum-Ileal vessel
pH	from 6 (T0) to 2.1	maintained at 6.8
Volume (mL)	50	90
Secretions	(**i**) 5.36 mg pepsin (727 U.mg^−1^) (**ii**) 4.28 mg lipase (32 U.mg^−1^) (**iii**) HCl 0.3 M (**iv**) NaHCO_3_ 0.5 M if necessary	(**i**) 0.9 g bile salts (27.9 mM in solution) (**ii**) 1.8 g of pancreatin 4 USP (**iii**) Trypsin 2 mg.mL^−1^ (**iv**) NaHCO_3_ 0.5 M if necessary
Time period in batch (min)	30	60
Chyme mixing	100 rpm (magnetic stirrer)	100 rpm (magnetic stirrer)
Concentration of Total microbes	sterile	sterile
Oxygen level (%)	20	20
Temperature (°C)	37	37

A static batch incubation (Erlenmeyer) was used to reproduce the physicochemical parameters of a gastro-ileal digestion according to parameters set-up in the TIM-1 system. Digestive secretions and solutions for pH adjustment were manually added during the 90 min digestion.

### Adhesion to mucin surface had a limited impact on ETEC virulence during gastrointestinal passage

**Fig. 3:**
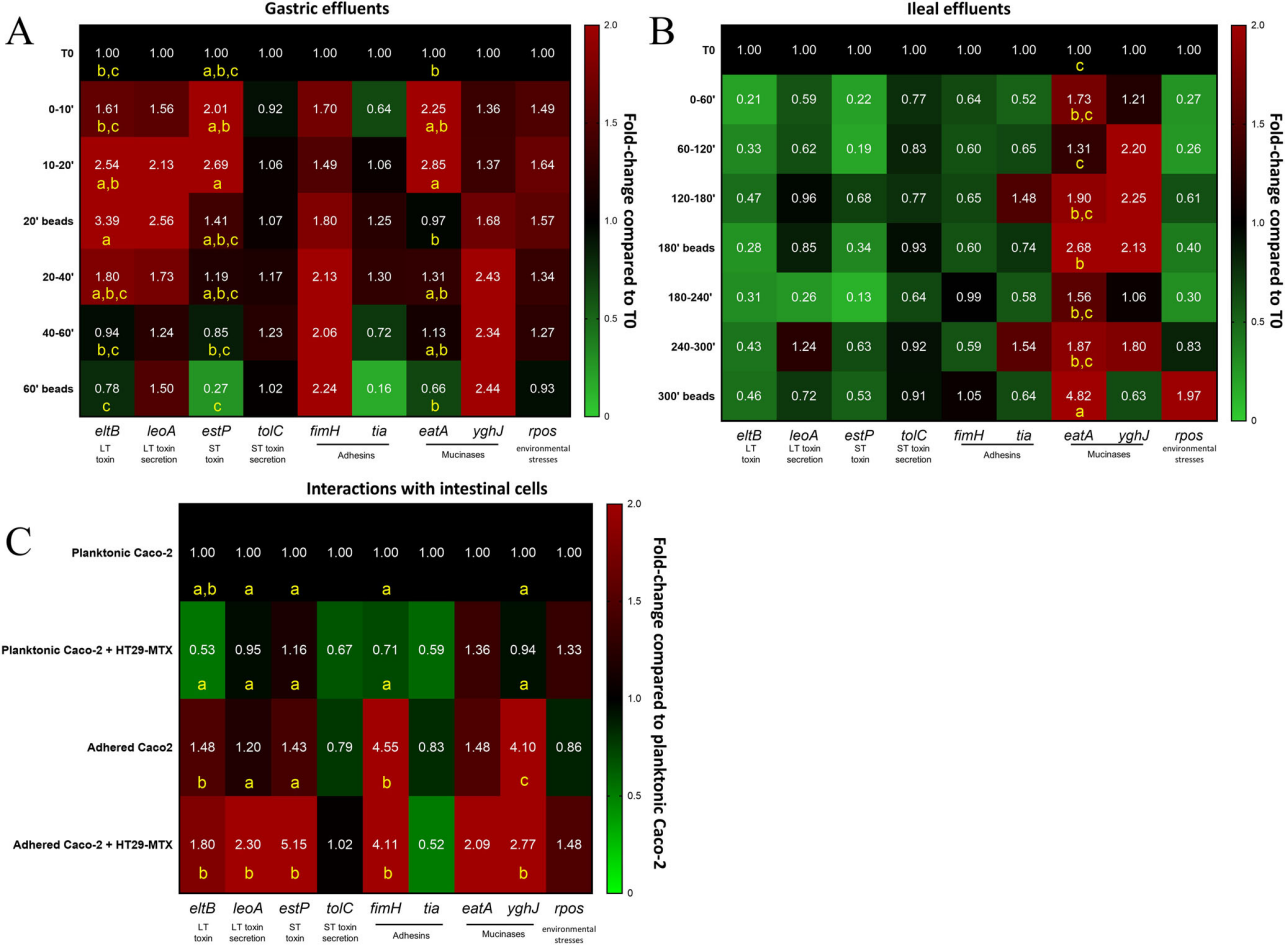
Dynamics of virulence gene expression in planktonic and adhered ETEC bacteria during gastrointestinal transit and interactions with intestinal cells. ETEC virulence gene expression was analyzed by RT-qPCR in the gastric (**A**) and ileal (**B**) effluents of the TIM-1 model inoculated with 10^10^ CFU and in cellular experiments (MOI 100) involving Caco-2 cells cultivated with or without HT29-MTX mucus-secreting cells (**C**). Gene expression was analyzed over-time in the TIM-1 on planktonic bacteria or bacteria adhered to mucin beads (**A**, **B**) or intestinal cells (**C**). Results were expressed and colored according to fold-change expression compared to ETEC gene expression in the glass of water used to inoculate the TIM-1 model (T0) (**A**, **B**) or planktonic bacteria upon Caco-2 cells (**C**). Assayed genes were *estP* (ST toxin), *eltB* (LT toxin), *leoA* (LT toxin output), *tolC* (ST toxin outpout), *tia* (adhesin), *fimH* (minor component of type I pilus), *yghJ* (mucinase), *eatA* (mucinase) and *rpos* (environmental stress response). Results that are significantly different from each other according to Tukey’s multi-comparison are grouped under different yellow letters (*p* < 0.05).

To assess how ETEC adhesion to a mucus surface could affect bacterial virulence during the gastrointestinal passage, we investigated the expression of 9 genes (Table 3) in the luminal planktonic ETEC and mucin-adhered bacteria ETEC in the TIM-1 model (Fig. 3). In the gastric compartment, ETEC strain H10407 virulence gene expression splits into two profiles dependent of the time. Excepted the genes *fimH* and *yghJ* A, global increase of gene expression was noticed until 40 min digestion (up to 2.9- fold) while gene expression returned to a baseline at the end of gastric digestion (Fig. 3A). The overexpression of *eltB* in beads-adhered bacteria at 20 min and *eatA* in planktonic bacteria between 10 and 20 min reached significance (*p* < 0.05). Globally, adhesion to mucin beads had a minor impact on ETEC-associated virulence in the stomach (Fig. 3A). No statistical difference had been observed except for *eatA* gene, since its expression was decreased at 20 min on mucin beads compared to luminal bacteria between 10 and 20 min (*p* < 0.05). In the ileal effluents, virulence gene expression was globally repressed all along the course of digestion, except for *eatA* and *yghJ*, the two ETEC mucinase genes, with around a twofold increase in expression (Fig. 3). In particular, the up-regulation of *eatA* gene reached significance on mucin beads at both 180 and 300 min, with respectively 2.7- and 4.8-fold increases compared to the initial inoculation level (*p* < 0.05). The effect of adhesion on mucin beads was subtle with a significant 2.6-fold increase for *eatA* in adhered bacteria at 300 min when compared to the luminal ones (*p* < 0.05).

### Adhesion to mucus-secreting cells favored ETEC virulence gene expression

To decipher the role of the mucus microenvironment in ETEC virulence during host interactions, virulence gene expression was further measured on both planktonic and adhered bacteria during infection of Caco-2 or Caco-2/HT29-MTX cells (Fig. 3C). Whatever the cell model (mono- or co-culture), the adhesion of ETEC was associated with a global expression increase of most of the virulence gene invetigated (Fig. 3C). When considering the monoculture model only, the expression of *fimH* (4.6 fold) and *YghJ* (4.1 fold) increased significantly with adhesion (*p* < 0.05). In the co-culture model, significance was reached with upregulation of *eltB*, *leoA*, *estP*, *fimH* and *yghJ* genes in adhered cells compared planktonic ones (3.7-, 2.4-, 4.4-, 5.8- and 3.0- fold, respectively, *p* < 0.05). When focusing on the adhered bacteria populations, with exception of *yghJ*, the expression of all virulence genes assayed was higher in the Caco-2/HT29-MTX mucin-secreting model compared to the Caco-2 nonmucin-secreting model. These increases reached significance for *leoA* and *estP* (*p* < 0.05) (Fig. 3C). Inversely, when focusing on planktonic bacteria only, the expression for most virulence genes, except *estP* and *eatA*, tended to decrease with the co-culture when compared to the monoculture, yet without reaching significant differences (Fig. 3C). Hence, the combination of cellular adhesion and the presence of mucus-secreting cells were associated with an increased expression of ETEC virulence genes.

### Mucus-secreting cells contributed to ETEC IL-8 induction

**Fig. 4:**
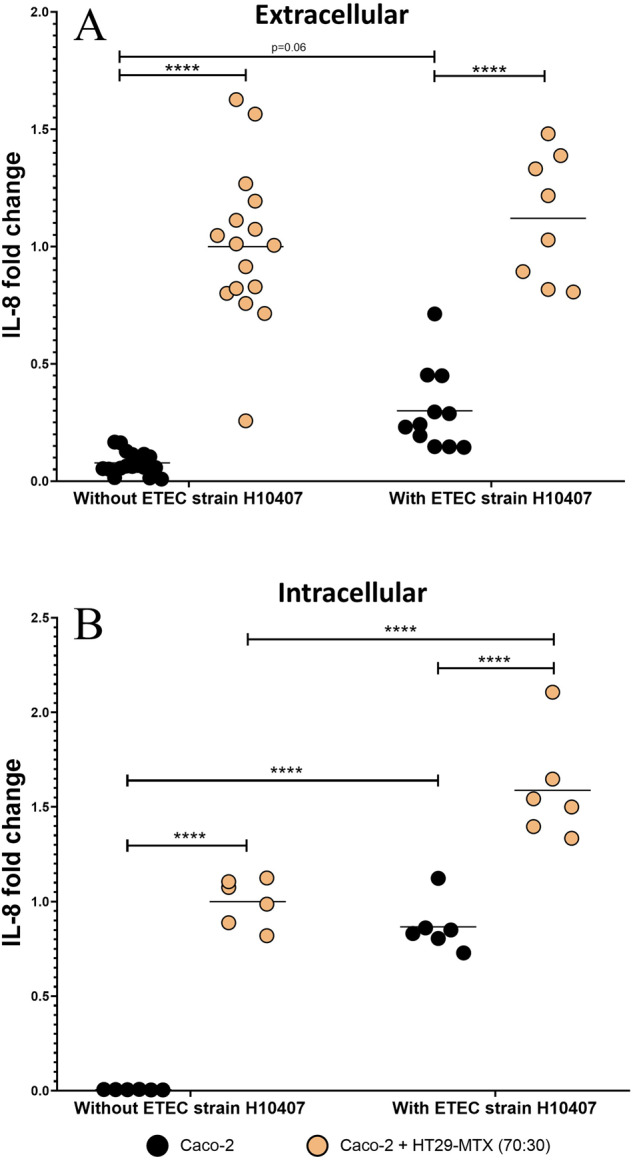
ETEC induction of Interleukin-8 production by mucin secreting or non-secreting intestinal cells. Interleukin-8 (IL-8) extracellular secretion (**A**) and intracellular production (**B**) by ETEC-infected Caco-2 (black dots) or Caco-2/HT29-MTX (orange dots) cells were measured by an ELISA assay. Intestinal cells were infected for a 3-hour period with 10^7^ CFU.mL^−1^ (MOI 100) with ETEC strain H10407. Control experiments were performed without the bacteria. Results are expressed as fold changes compared to non-infected Caco-2/HT29-MTX cells. The data represents the replicates of at least 3 independent experiments with their means (black line). Statistical differences provided by Tukey’s multiple comparisons test are indicated (****p < 0.0001).

To assess whether adhesion and virulence gene expression associated with the mucus microenvironment exacerbated ETEC induced-inflammation, pro-inflammatory IL-8 cytokine was measured prior and following infection in cell assays (Fig. 4). ETEC infection was associated with a significant rise in IL-8 level, but only for intracellular production (*p* < 0.001). Whatever the infection status (infected or non-infected cells), co-culture with mucin-secreting cells led to a significant increase in extracellular (Fig. 4A) and intracellular (Fig. 4B) IL-8 levels compared to Caco-2 alone (*p* < 0.001). After a 3-hour infection period with ETEC, intracellular IL-8 levels significantly increased by 1.8-fold from monoculture to co-culture conditions (*p* < 0.001). Thus, addition of mucus-secreting cells did not impede the induction of IL-8 production and even increased IL-8 intracellular levels.

### Mucin surface did not favor ETEC colonization in a complex microbial background

**Fig. 5:**
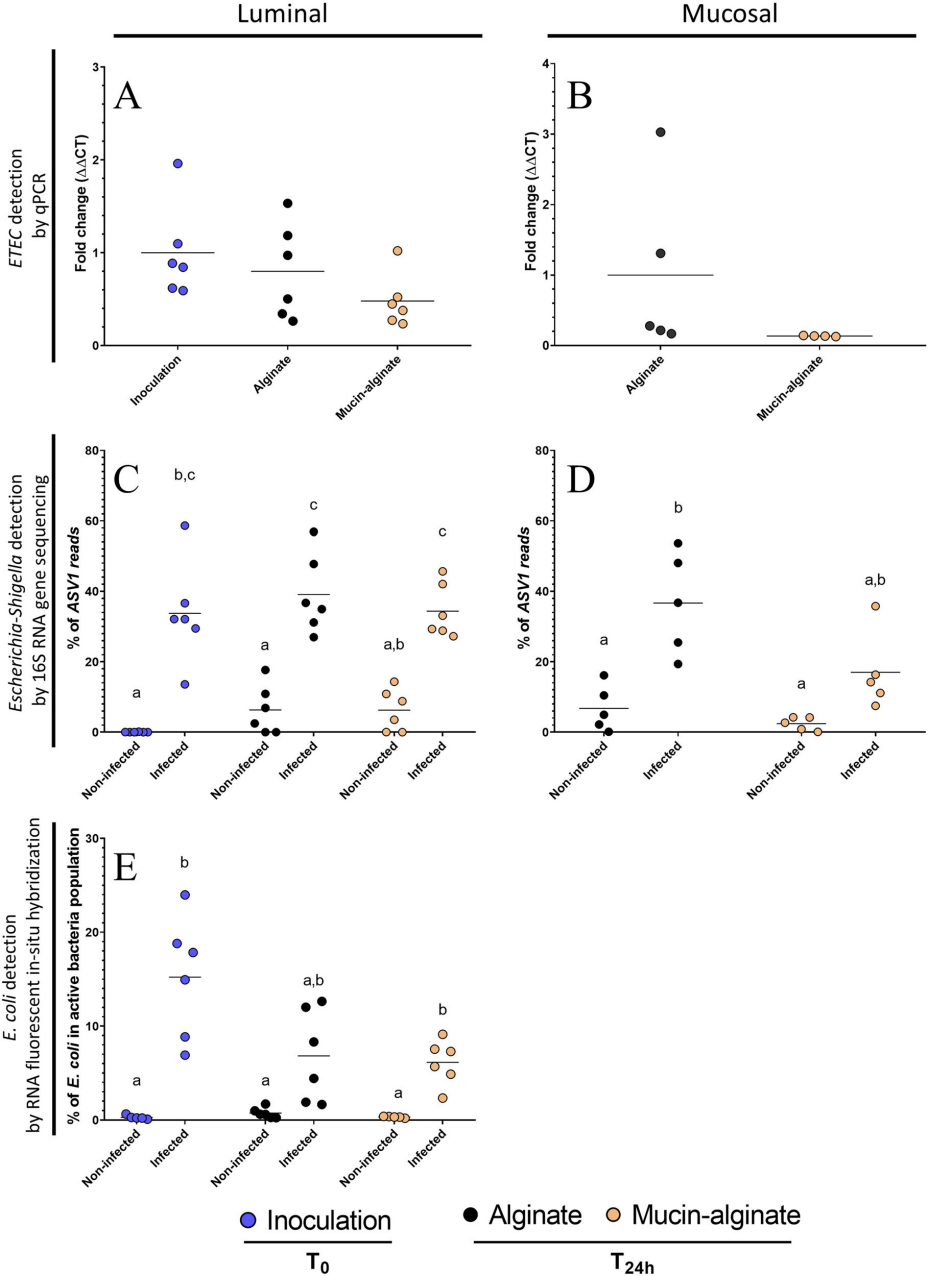
Impact of mucin on ETEC survival in in vitro fecal fermentation batches. Penicillin bottles with mucin-alginate beads or alginate beads (as a control) were inoculated with feces from 6 healthy donors and challenged or not with pre-digested ETEC strain H10407 at 10^8^ CFU.mL^−1^. Blue, black and orange dots represent individual biological replicates at the beginning of the experiment after ETEC inoculation (inoculation T0) or after 24 h fermentation with alginate (alginate T24) and mucin-alginate (mucin-alginate T24) beads, respectively. **A, B** qPCR detection of ETEC strain H10407 among total bacterial populations expressed as fold changes compared to inoculation T0 (luminal phase) or alginate condition (mucosal phase). **C, D** Percentages of ASV1 reads detected by 16S rRNA gene amplicon sequencing in planktonic and adhered ETEC bacteria. ASV1 is the ASV with the highest reads abundance in all samples and its reads have been assigned to the *Escherichia*/*Shigella* genus and to *Escherichia albertii/boydii/coli/dysenteriae/fergusonii/flexneri/marmotae/sonnei* species. **E** Proportion of active *E. coli* in the total bacterial populations as detected by RNA fluorescent in situ hybridization. Black bars represent the mean of data (*n* = 6). Results that are no significantly different from each other according to Tukey’s multi-comparison are grouped under the same letter (*p* < 0.05).

To investigate how a mucus physical surface may impact ETEC interactions with human fecal microbiota, batch experiments inoculated with human feces were conducted by addition of mucin-alginate beads or alginate beads as a control (Fig. 5). ETEC as well as *E. coli* taxa were investigated using high-throughput analyses. As expected, after inoculation, *E. coli* taxa became predominant in the luminal phase of infected bottles and represented on average 34% of the total detected bacterial ASVs (amplicon sequence variants) reads by 16S rRNA gene sequencing (Fig. 5C) and 15% of the total active bacteria assayed by RNA fluorescent in situ hybridization (Fig. 5E). According to quantitative PCR (qPCR) and 16S rRNA gene sequencing data, under both conditions (alginate and mucin-alginate beads), ETEC strain H10407 and *Escherichia-Shigella* taxa did not significantly decrease in the luminal phase of ETEC-infected bottles during the experiment (Fig. 5A, C), while RNA flow-FISH showed a non-significant 2-fold decrease of the *E. coli* active population (Fig. 5E). Whatever the molecular analytical technique used, mucin did not impact ETEC survival nor *Escherichia* abundance after a 24-hour fermentation period (Fig. 5A, C, E). Even if no significance was reached due to important donor variation, results were more striking in the mucosal niche. According to qPCR results at T24h, ETEC level tended to be on average 7.4-fold lower in the mucin-alginate beads compared to alginate conditions (Fig. 5B). Accordingly, the proportion of *Escherichia-Shigella* population tended to be 2.2-fold lower on mucin-alginate beads according to 16S rRNA gene sequencing analyses (Fig. 5D). Hence, in opposition with the results obtained in the TIM-1 model in the absence of resident microbiota (Fig. 2), the inclusion of mucin-alginate beads in fecal batch experiments did not favor an over-representation of ETEC (Fig. 5).

### Mucin-associated microbiota was particularly affected by ETEC colonization

**Fig. 6:**
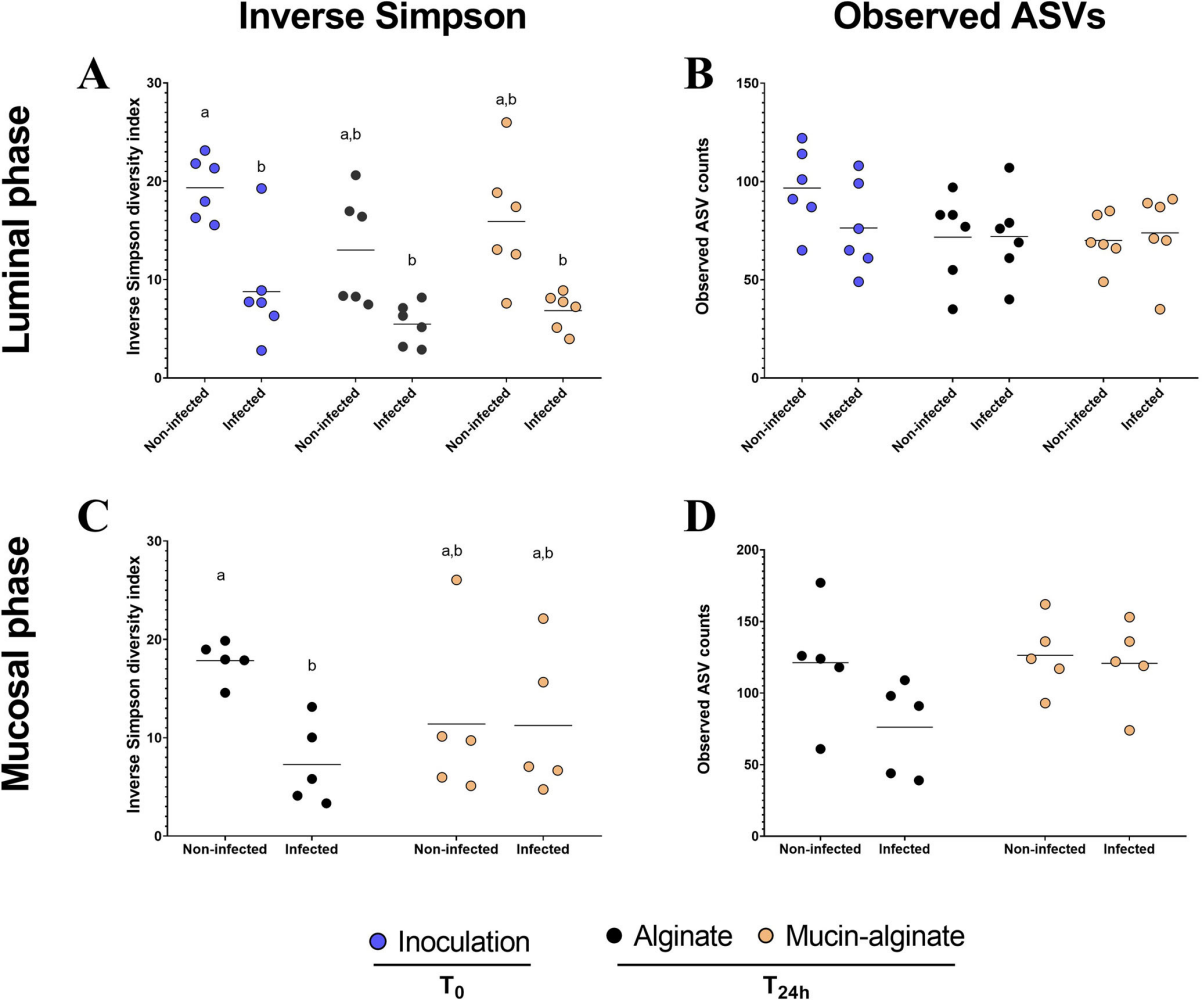
Impact of mucin on ETEC modulation of fecal microbial communities as determined by α-diversity. Batch experiments were performed using feces from 6 healthy donors, challenged or not with ETEC strain H10407, with mucin-alginate beads or alginate beads (as a control). The graphs represent the variation of the microbiota α-diversity at the ASV level at inoculation (T0) and after 24 h (T24h) between bottles including mucin-alginate beads and alginate beads in luminal (**A**, **B**) and mucosal compartments (**C**, **D**). The parameters analyzed included species richness represented by Observed ASVs (**B, D**) and evenness represented by Inverse Simpson index (**A**, **C**). Blue, black and orange dots represent individual biological replicates at the beginning of the experiment (T0) or after 24 h (T24h) in the alginate and mucin-alginate beads conditions, respectively, while black bars represent the mean. Results that are not significantly different from each other according to Tukey’s multi-comparison are grouped under the same letter (*p* < 0.05).

**Fig. 7:**
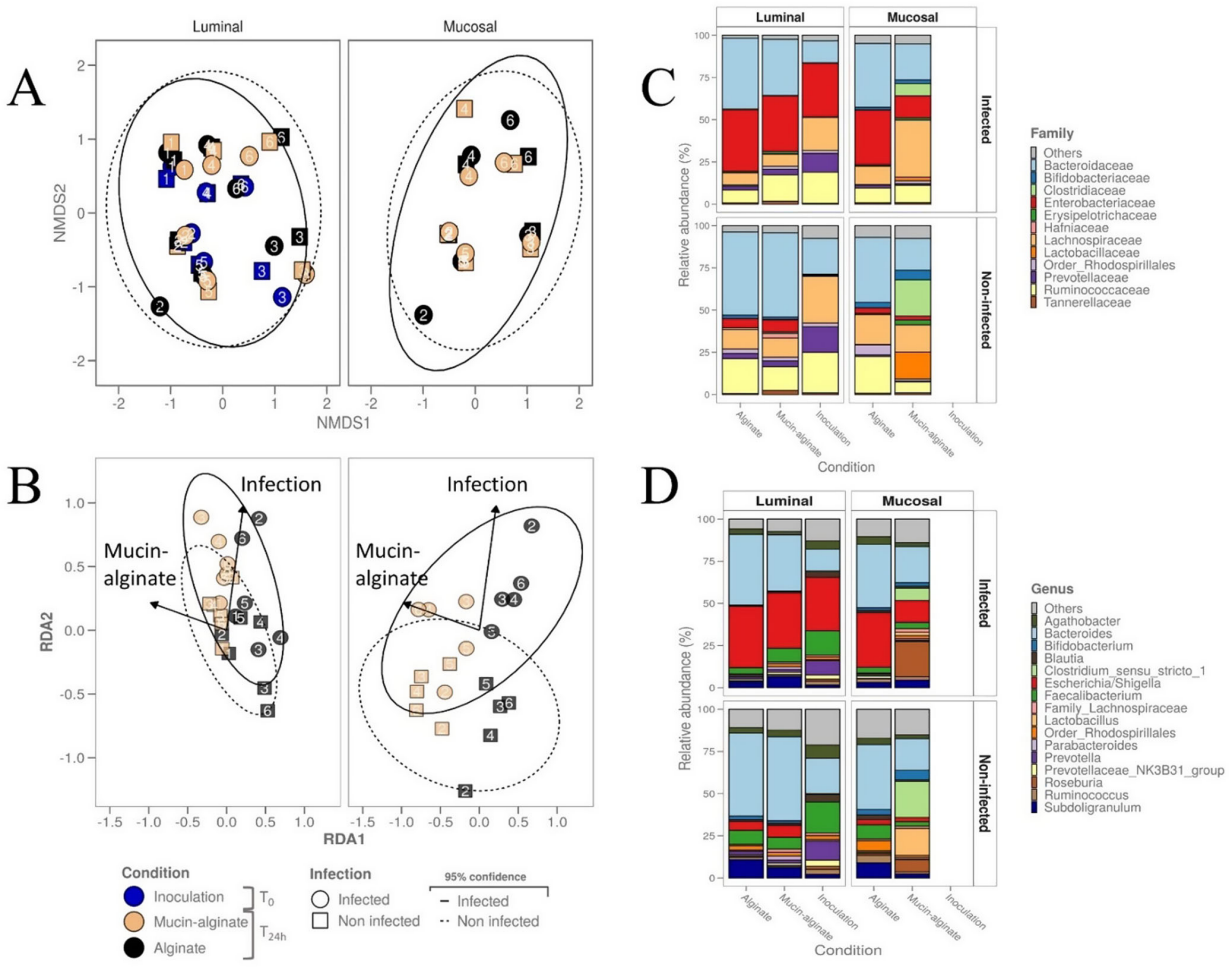
Impact of mucin on ETEC modulation of fecal microbial communities as determined by β-diversity. Batch experiments were performed using feces from 6 healthy donors, challenged or not with ETEC strain H10407, with mucin-alginate beads or alginate beads (as a control). **A** Non-metric multidimensional scaling (NMDS) and (**B**) distance-based redundancy analysis (db-RDA) two-dimension plot visualization report the microbial community β-diversity at the ASV level, as determined by 16S rRNA gene amplicon sequencing. For db-RDA, ASV1 (attributed to *Escherichia*/*Shigella* genus) was excluded from the relative ASV table and “infection” and “mucin” were provided as sole environmental variables (binary) and plotted as vectors (arrows). Blue, black and orange dots represent individual biological replicates (donor numbers are indicated) at the beginning of the experiment (inoculation, T0) or after 24 h of fermentation (T24h) with alginate and mucin-alginate beads, respectively. Samples are represented by dot shape and square shape for the infected and non-infected conditions, respectively. The 95% confidence ellipse area is also indicated in continuous line for the infected condition and in dotted line for the non-infected condition. Cumulative bar plots of the relative microbial community composition at the family (**C**) and genus (**D**) levels. The area graphs show the relative abundance of the 12 most abundant families and 16 most abundant genera with all six different donors confounded.

To further explore how a mucus surface would modulate ETEC impact on fecal microbiota composition, we performed Illumina 16S rRNA gene amplicon sequencing and bacterial community analysis. Concerning α-diversity, ETEC inoculation tended to reduce bacterial species evenness in the luminal compartment regardless of the tested condition (Fig. 6). Notably, the Inverse Simpson index tended to be reduced following ETEC challenge by 2.2-, 2.4- and 2.3- fold at inoculation (T0) and 24 h post-infection with alginate and mucin-alginate beads, respectively (*p* < 0.05) (Fig. 6A). Whatever the index considered (Shannon, Simpson, Inverse Simpson or observed ASVs counts), addition of mucin did not impact the α-diversity (Fig. 6A, B, Supplementary Fig. 2). Results are more striking in the mucosal niche. In the control condition (alginate beads), ETEC inoculation reduced species evenness (2.4-fold decrease for the Inverse Simpson index, *p* < 0.05, Fig. 6C) and tended to reduce richness (1.6-fold decrease of observed ASVs, Fig. 6D). Interestingly, these decreases were not observed with mucin-alginate beads (Fig. 6C, D). Regarding β-diversity, non-metric multidimensional scaling (NMDS) analysis showed that microbiota origin is the predominant explanatory variable for dissimilarities in fecal microbiota composition, both in the luminal and mucus niches (Fig. 7A). Still, Permutational Multivariate Analysis of Variance (permANOVA) analysis conducted on the samples at T24h and excluding ASV1 (attributed to *Escherichia/Shigella*), reported that ‘infection’ and ‘mucin’ factors significantly accounted for 10.4% (*p* < 0.001) and 3.8% (*p* < 0.05, 999 permutations) of the dissimilarities, respectively (Supplementary Fig. 3). To go further, distance-based redundancy analysis (db-RDA) was performed using mucin condition and ETEC challenge (‘infection’) as explanatory variables. ASV1 (attributed to *Escherichia*/*Shigella* genus) was also excluded from the analysis to efficiently capture the impact of different conditions towards the gut microbiota community (Fig. 7B). The db-RDA was able to cluster more efficiently samples from mucin condition *versus* alginate condition in the mucus niche (Fig. 7B, Supplementary Fig. 3), indicating that the effect of mucin on fecal microbiota composition was greater on the mucus-associated microbiota than on the luminal one. Non-infected mucin beads display a specific microbiota that was particularly enriched in *Clostridium*, *Roseburia* and *Lactobacillus* ASVs (Fig. 7C, D, Supplementary Fig. 4), even if *Lactobacillus* colonization appeared to be donor-dependent (Supplementary Figs. 5 and 6). ETEC infection tended to specifically influence this mucin-associated microbiota, associated with decreases in *Clostridium, Lactobacillus* and *Bifidobacterium* and an increase in *Roseburia* ASVs (Fig. 7C, D and Supplementary Fig. 5). In this sense, the mucus phase of mucin-alginate beads was the only condition for which ASVs were found to be significantly modulated by ETEC infection (Supplementary Fig. 7). Overall, these results support a specific impact of ETEC infection on the β-diversity of mucin-beads associated bacterial communities.

### Mucin had a modest impact on fecal microbial activities

**Fig. 8:**
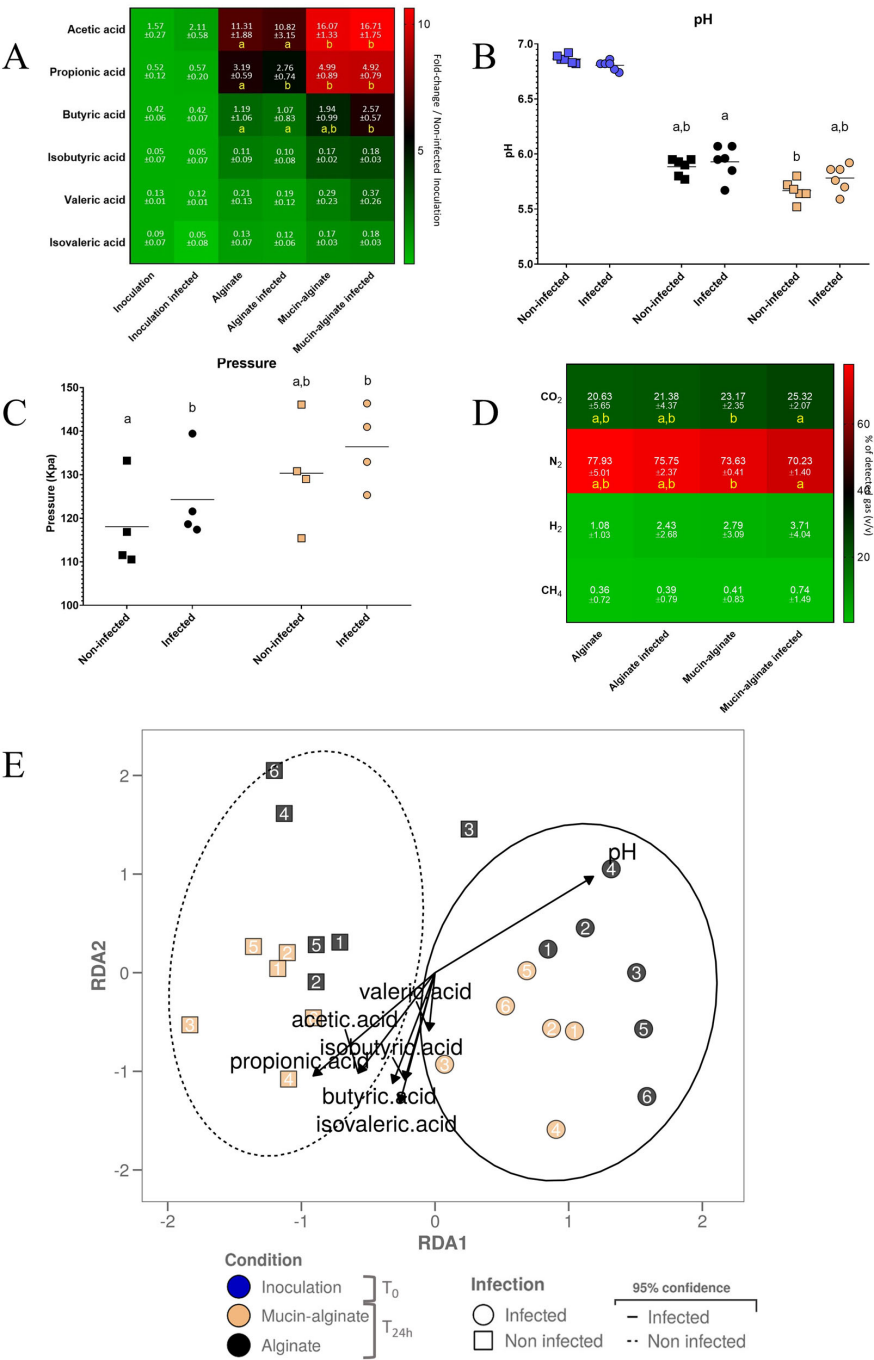
Impact of mucin on ETEC modulation of fecal microbial activity. The impact of ETEC inoculation (infected *versus* non-infected) and mucin (mucin-alginate *versus* alginate beads) on gut microbiota activity in batch fermentations were assayed by the measurement of SCFAs production (**A**), pH acidification (**B**), gas pressure (**C**) and gas composition (**D**). Experiments were performed using fecal samples from 6 healthy donors. Blue, black and orange dots represent samples collected at the beginning of the experiment (inoculation, T0) or after 24 h fermentation (T24h) with alginate and mucin-alginate beads, respectively. (**A**) SCFA production in the luminal phase was analyzed by liquid chromatography. Results were expressed in mmol (*n* = 6) and colored according to fold change compared to the control condition (non-infected, T0). (**B**) pH of the fermentation medium was recorded over-time at T0 and T24h and biological replicates are represented as dots with their means (black line). (**C**) Gas pressure was measured at T24h and biological replicates are represented as dots with their means (black line). (**D**) Gas composition was determined by gas chromatography at T24h. Results were expressed as mean percentages ± standard deviation (*n* = 6) and accordingly colored. (**E**) The 2 dimensions-plot reports the β-diversity structure of the whole microbial community taxonomy at the ASVs level in the luminal phase according to db-RDA according to metabolites variables (namely SCFAs and pH). Individual samples are represented by dot and square shapes for the infected and non-infected conditions, respectively. The 95% confidence ellipse zone is also indicated in continuous line for the infected condition and in dotted line for the non-infected condition. The donor number is indicated for each sample. Results that are not significantly different from each other according to Tukey’s multi-comparison are grouped under the same letter (*p* < 0.05).

In a last step, the effect of mucin on human gut microbial activity during ETEC infection was assessed by following various indicators such as short chain fatty acids (SCFAs), gas production, pH acidification and gas pressure (Fig. 8). Compared to control alginate beads, mucin-alginate led in non-infected condition to a significant increase in the amount of SCFAs, as reported by the levels of acetic (1.4-fold increase) and propionic (1.6-fold increase) acids (*p* < 0.05). Under ETEC-infected conditions, mucin addition led also to a significant increase (*p* < 0.05) in acetic, propionic, and butyric acids by 1.5, 1.8, and 2.4-fold, respectively (Fig. 8A). Regarding pH acidification, addition of mucin-alginate beads resulted in a lower pH at T24h of fermentation compared to the control beads, in both non-infected and ETEC-infected bottles (*p* < 0.05). Surprisingly, the infection by ETEC tended (*p* = 0.08) to limit the pH decrease at T24h regardless of mucin presence (Fig. 8B). Interestingly, ETEC infection induced a significant increase in gas pressure with control alginate beads (*p* < 0.05). Gas pressure was also influenced by mucin addition, with a non-significant 10% increase in both infected and non-infected conditions (Fig. 8C). Regarding gas production (Fig. 8D), ETEC infection induced significant changes when mucin was added, as shown with increasing CO_2_ percentage by 2.1% while decreasing N_2_ level by 3.4% (*p* < 0.05). Mucin presence also influenced headspace gas profiles, by increasing both CO_2_ and H_2_ and decreasing N_2_. Yet, a mucin-dependent significant effect was not reached due to high donor variabilities. To further investigate how microbial metabolite production could be associated to changes in microbial community structure, ‘SCFA’ and ‘pH’ were included as explanatory variables in a db-RDA analysis performed on the whole ASVs table of the luminal samples. Samples were then clustered according to ‘infection’ and ‘mucin’, proving that these variables accounted for some of the differences in β-diversity taxonomy structure between tested conditions (Fig. 8E). Supporting the data presented in this section, pH increase correlated with the taxonomy structure of the alginate beads and infected samples, while SCFAs production only correlated with the mucin bead taxonomy structure. Thus, ETEC infection and the type of beads modulated gut bacterial activity in a modest but significant manner.

## Discussion

The particular relationship between enteric pathogens and the mucus layer, which represents both a physical barrier, an anchorage surface to adhere to and a possible nutrient source, is currently underexplored. Using complementary in vitro models of the human lumen and cellular digestive environment, we showed the key role of the mucus microenvironment in ETEC H10407 survival, adhesion, virulence and interactions with the human fecal microbiome. For this purpose, we used porcine gastric mucin, which exhibits acknowledged differences with the human intestinal mucin^44,45^, but is the only commercially available source allowing us to obtain the amount of materials needed for our experimental set-ups. Similarly, Caco-2/HT29-MTX cells is one of the most frequently used co-culture model, even if the mucus composition/structure does not fully reflect the in vivo situation. In particular, its mucus is essentially composed of MUC5 and forms patches rather than a proper continuous layer^46–52^.

### ETEC survival throughout the gastrointestinal tract

Low gastric pH is the first challenge ETEC faces upon ingestion^53^. In this environment, the addition of mucin did not seem to reduce pathogen mortality overtime in the luminal compartment, despite its obvious buffering effect on the gastric pH. However, such phenomenon is associated with a lower fraction of ETEC exposed to lethal pH values in the stomach, resulting in more viable bacteria reaching subsequently the duodenum (significant at T60 min). The next hurdle during gastrointestinal passage is the release of bile salts in the duodenum, with a well-known deleterious effect on enteric pathogen survival by disrupting bacterial membranes^54,55^. In accordance with previous studies performed in the TIM-1 on various *E. coli* pathotypes^18,56,57^, ETEC strain H10407 viability dropped in the duodenal compartment. Nevertheless, mucin addition led to a slight increase in viable bacteria from 120 min to the end of the duodenal digestion. Then in the distal part of the small intestine, less stringent conditions (i.e. pH close to neutrality, lower bile salts concentrations due to re-absorption and/or longer residence times) allowed ETEC to grow, as previously shown for other *E*. *coli* strains^18,56,58^. This was particularly striking with mucin, with an exponential increase in cell concentration in both the jejunal and ileal compartments of TIM-1. Such growth can certainly be explained by the presence of nutrients brought by mucin and supported by the capacity of ETEC strain H10407 to grow on minimal culture medium supplemented with type III mucin. It is likely that release of mucin-derived sugars could represent an important reservoir of nutrients that promotes the growth of ETEC as previously shown in vitro for other pathotypes^59,60^. Lastly, ETEC survival was evaluated during short-term in vitro batch incubations as a simple model of human colonic conditions. Even if the pathogen action site is generally considered to be the distal part of the small intestine^19,21,61–64^, the shedding after infection remains particularly important^65,66^ and studying survival in an environment where the gut microbiota prevails in high number is undoubtedly relevant. Some authors already performed fecal fermentation experiments with human ETEC strains in in vitro models, including a mucus compartment (mucin-agar microcosms), both in batch experiments, yet with a poor microbial background simulating “dysbiotic” condition^42^, and in the multi-compartmental Simulator of the Human Intestinal Microbial (SHIME) model^18^. Nevertheless, they did not address specifically this mucus microenvironment impact on ETEC survival. Here, we report that ETEC and/or *E. coli* are able to maintain their relative presence during 24 h in normally-inoculated batch experiments (i.e. normal microbial background). The inclusion of mucin beads did not significantly impact ETEC survival in the luminal compartment. We argue that the mucin-derived substrates released in the luminal phase are negligible compared to the nutrients supplied by the batch medium, and therefore have less impact on these parameters, as evidenced by microbiota composition results. Overall, our results concerning ETEC survival support that the pathogen could use mucus to better cope with the stressful upper gastrointestinal conditions or to multiply in the lower intestinal compartments, but only when no other nutrient source is available.

### ETEC adhesion on the mucus compartment

Besides survival in the luminal environment, adhesion to mucus as a physical surface is a next challenge for pathogens to colonize the gastrointestinal tract. The high percentages of ETEC associated to mucin-alginate beads in the TIM-1 stomach and duodenum at the end of digestion indicated that the presence of a mucus surface constitutes a significant protective micro-niche enabling longer survival under stringent conditions (e.g. acidic pH, bile acids), with specific physicochemical-parameters different from the lumen^4^. In vivo, gastric mucus is already well-known to harbor a pH gradient protecting the epithelium from the acidic pH^67^. It could be envisioned that gastric mucin polymers have the ability to sequestrate proton^68^. *Helicobacter pylori* has been previously described to benefit from this mucus layer shelter to maintain its presence in the stomach^69,70^. The present study suggests that this concept might be extended to the survival of pathogens that display a more distal tissue tropism in the upper gut. Lastly, in colonic batch experiments, we reported that ETEC gene copy numbers tended to be lower on mucin-alginate beads compared to control beads, particularly in the presence of certain individual donor microbiota. This results suggest that in a complex microbial background, specific colonization of mucin beads by the endogenous microbiota could certainly protect from ETEC engraftment in a donor-dependent manner. Of note, due to the simplicity of the batch models, which do not reproduce the renewal of nutritive medium, we cannot fully appreciate whether the beads act as a colonization reservoir for the lumen as mucus does in vivo. To go further, additional experiments should be performed in more complex continuous fermentation systems^18^.

Since access to the mucin surface was shown to be meaningful for the pathogen survival during gastrointestinal transit in the TIM-1 system, the specificity of ETEC adhesion to mucus compartment was therefore investigated using different in vitro assays, including control conditions without mucin. A significantly higher adhesion of ETEC strain H10407 was shown on mucin-alginate compared to alginate beads after a simple gastro-intestinal digestion procedure, as well as an increased adhesion to mucus-secreting cells compared to non-secreting ones. Our results are in accordance with previous studies showing that in vitro adherence of *Salmonellae enterica* serovar Typhimurium and Enterohemorragic *E. coli* (EHEC) was higher on high-mucin producing cells (e.g. HT29-MTX or LS174T) than in non- or low-mucin producing cells (e.g. Caco-2 or HT29)^71,72^. All together these data suggest that pathogens belonging to the *Enterobacteriaceae* family are well adapted to the intestinal mucus barrier. Such idea was strengthened by a recent study showing that Enteroaggregative *E. coli* (EAEC) and EHEC specifically adhere to mucus droplets in human enteroids^73^. Of note, Kerneis and colleagues showed that ETEC strain H10407 binding on HT29-MTX cells did not necessarily co-localize with mucus, suggesting that the strain affinity could also be due to the recognition of HT29-MTX surface receptors^74^.

### ETEC virulence gene expression

To achieve its infectious cycle, ETEC has to efficiently express a large panel of virulence genes, especially those related to toxin secretion. In simple broth media, mucin has already shown to influence virulence and motility of pathogens such as *Campylobacter jejuni* and EHEC^75,76^ and concerning ETEC, to support CFA/I and CS1/CS3 colonization factor expression but to decrease LT toxin secretion^44^. By incorporating mucin beads in the TIM-1 model, we were able to investigate the impact of adhesion to a physical mucosal surface on ETEC virulence expression in a relevant model of the human upper gut. Our results showed that the impact of mucin-beads adhesion on ETEC virulence was quite subtle, indicating that adherence to a mucin surface has a poorer influence on ETEC strain H10407 virulence regulation compared to changes in digestive physicochemical parameters during gastrointestinal passage, as previously shown by Roussel et al.^18^. This modest effect of adhesion can be due to the presence of mucin in the luminal phase of the TIM-1, added to accurately simulate mucus constant shedding. Using cellular culture approaches, we further investigated how host-bacteria interactions modulate ETEC virulence. Cell adhesion was strongly associated to virulence gene expression, even more with mucus-secreting cells. Our results are in accordance with a study from Kansal and colleagues showing that cell contact enhanced transcription of LT and Type 1 pilus encoding genes in ETEC strain H10407^77^. It is noteworthy that the same authors found that another ETEC strain decreased its virulence with cellular proximity, indicating that such data are strain-specific^77^. The greater impact of mucus-secreting cells on ETEC virulence could be attributed to specific glycoproteins secreted by HT29-MTX. In particular, HT29-MTX cells mainly secrete MUC5AC and MUC5B mucin^46–48^. Pig gastric mucin is also mainly composed by MUC5AC and MUC5B^44,45^ and has been found to positively influence colonization factor (CF) expression in various ETEC strains including H10407^44^.

### IL-8 ETEC induction

We next considered if addition of mucus-secreting cells, for which ETEC strain H10407 has an obvious adhesion affinity, could result in changes in pro-inflammatory IL-8 levels. Despite an increased basal production in the co-culture model, we were able to measure significant IL-8 induction by ETEC infection. This is in line with previous studies showing that ETEC toxins and YghJ mucinase induce cellular inflammation^36,37,78^, as depicted in our study with all the associated genes being overexpressed by adherent bacteria in the co-culture model. These results also suggest that the mucus layer (or rather mucus patches phenotype)^49–51^ in HT29-MTX cells does not sufficiently decoy the bacteria from the epithelial close contact to inhibit IL-8 induction. Our results might be challenged by a recent study on human intestinal organoids, a model that more accurately reflects the human gut epithelium than the Caco-2/HT29-MTX coculture, that ETEC does not stimulate IL-8 secretion^79^.

### ETEC impact on microbiota composition

Several studies have already demonstrated the impact of human ETEC strains on fecal microbiota composition in vivo^39–41^. As gut microbiota alterations could modulate infections outcomes^80,81^, it is crucial to better decipher the impact of such changes on the infection process. As such, we investigated the combined modulatory effects from the presence of a mucus microenvironment and ETEC presence towards human fecal microbiota. ETEC infection was associated with a bloom of *Escherichia*/*Shigella* (most probably ETEC), a decrease in gut microbiota evenness, and a modest impact on β-diversity which is a confirmation of previous observations in humans^39,40,82^. The addition of mucin-alginate beads had a minor impact on α and β-diversities indices in batch colonic incubation in the luminal compartment. However, we reported the colonization of mucin beads by a specific microbiota characterized by increase in *Clostridium* and *Bacillus* species, as previously shown in the mucosal compartment of the SHIME model^83,84^. This specific microbiota might be responsible for the inhibition of the observed ETEC colonization and the maintenance of α-diversity on mucin beads. We also evidenced that specific mucus-associated microbiota was particularly impacted by ETEC inoculation. Given the known health-related properties of some impacted phylogroups (e.g. *Clostridium, Lactobacillus* and *Bifidobacterium*)^85–87^ and the clear association between mucosal microbiota in health and diseases^4^, the impact of ETEC challenge on the mucosal microbiota would deserve further investigations. It remains to be assessed whether the colonic mucus layer associated with a complex microbiota would contribute to enhance pathogen susceptibility or how this effect might vary between individual microbial communities in large cohorts.

### ETEC impact on microbiota activity

To date, few studies have focused on ETEC impact on fecal microbiota-derived metabolites. In fecal batch experiments, Moens and colleagues reported a decrease in SCFAs with ETEC infection^42^, while Roussel and colleagues showed an increase in propionic acid production in the M-SHIME model^18^. Here, we reported that ETEC infection increases gas production (increased pressure and CO_2_ level) but also limits the pH drop associated with fermentation activity. We argue that this feature could be due to *E. coli* acid resistance systems which notably consume H^+^ to produce H_2_O, H_2_ and CO_2_^88^. Unsurprisingly, we reported that the use of mucin beads, rich in nutritive substrates, resulted in increased production of fermentation end products. More interestingly, mucin beads seemed to boost ETEC impact on microbiota activity (higher level of CO_2_). This could be due either from higher requirement for acid resistance to counterbalance fermentation acidification or from ETEC mucinases activity leading to higher availability of substrates for commensal bacteria.

In conclusion, using a set of different but complementary in vitro devices of the digestive lumen and host intestinal cells, our integrated approach covering different intestinal conditions sheds more light on the dynamics of ETEC strain H10407 reference strain interacting with the mucus microenvironment in a human-related context. The mucus niche is usually and accurately seen as an efficient barrier against pathogenic invaders^13,89^. Supporting this view, we showed that the presence of a mucus-specific microbiota might be an effective mean against ETEC mucosal colonization in human-simulated colonic conditions. In this work, we also reported some ETEC pathophysiological features where the mucus presence does not necessarily represent an advantage for the host. Taken together, our findings propose that the presence of a mucus niche in the simulated upper gastrointestinal conditions favors ETEC survival in the digestive lumen and its adhesion to physical surface, thereby increasing the pathogen’s resilience against the harsh conditions of gastrointestinal passage. Adhesion to mucus-secreting intestinal cells also led to a sharp increase in virulence gene expression. Thus, we can argue that ETEC strains may have adapted to this mucus barrier and to some extent benefit from it. Further complementary studies in advanced in vitro models such as organoids and/or animal experiments are needed to confirm these promising in vitro results. These projects should open avenues to better understand the role of mucus in ETEC physiopathology and be paramount to develop new strategies to fight against these infections in humans.

## Methods

### ETEC strain and growth conditions

The prototypical ETEC strain H10407 serotype O78:H11:K80 (ATCC^®^ 35401, LT^+^, ST^+^, CFA/I^+^), isolated in Bangladesh from a patient with a cholera-like syndrome^90^, was used in this study. Bacteria were routinely grown under agitation (37 °C, 120 rpm, overnight) in Luria Bertani (LB) broth (Thermo Fisher Scientific, Waltham, MA, USA).

### Growth kinetics in M9 culture medium

ETEC strain H10407 (initial concentration of 10^7^ CFU.mL^−1^) was allowed to grow under aerobic conditions (37 °C, 5 hours, 120 rpm), in M9 medium (minimal medium for *E. coli* pH 6.8)^91^, with or without 3 g.L^−1^ mucin from porcine stomach type II or III (Sigma-Aldrich, St. Louis, MO, USA). The medium was regularly sampled and plated onto LB agar for ETEC numeration (*n* = 3).

### Mucin-agar adhesion plate assays

Adhesion experiments were adapted from Tsilia et al. (2016) as previously described^92,93^. Briefly, mucin-agar consisted of 5% porcine stomach mucin-type II (Sigma-Aldrich, St. Louis, MO, USA) and 1% bacteriological agar (Sigma-Aldrich, St. Louis, MO, USA), with a pH adjusted to 6.8 to mimic human small intestinal pH. Six-well plates containing mucin-agar were inoculated with ETEC strain H10407 (initial concentration of 10^7^ CFU.ml^−1^). After 1-hour incubation (37 °C, 120 rpm), each well was rinsed twice with phosphate buffer saline (PBS) to remove non-adherent bacteria. Separation of adhered bacteria was mechanically performed by transferring aseptically the whole mucin layer into a sterile bag containing PBS and further homogenization in a 400 P BagMixer^®^ for 10 min (Interscience, Bread, Netherlands). Adhered ETEC bacteria were quantified by plating onto LB agar. Experiments were performed in triplicate and agar without mucin was used as a negative control.

### Mucin beads preparation

Mucin-alginate beads were obtained as already described^94^. The mixture containing 5% (w/v) porcine gastric type III mucin (Sigma-Aldrich, Saint-Louis, MO, USA) and 2% (w/v) sodium alginate (Sigma-Aldrich, Saint-Louis, MO, USA) was dropped using a peristaltic pump into a sterile solution of 0.2 M CaCl_2_ under agitation (100 rpm). Control beads with the same density, but without mucin were produced using alginate at a final concentration of 4.5%. Beads (diameter: 4.5 mm in average, data not shown) were then stored at 4 °C (no more than 24 h prior experiments).

### In vitro static and dynamic digestion procedures

Static in vitro gastro-jejunal digestions were performed before mucin-beads adhesion assay (50 beads were added in the duodenum-ileum vessel) or to simulate upper gastrointestinal stresses experienced by ETEC before colonic batch fermentation experiments (without mucin beads), as previously described^18^ (Table 1). For adhesion assays, ETEC strain H10407 was inoculated at 10^7^ CFU.mL^−1^ and experiments were performed in triplicate. Inoculation rates were calculated to ensure an initial concentration of 10^8^ CFU.mL^−1^ in batch experiments (*n* = 6).

Dynamic digestions were also performed using the TIM-1 system, which consists of four successive compartments simulating the human stomach and the three parts of the small intestine (duodenum, jejunum, and ileum). This in vitro system integrates the main physicochemical parameters of human digestion, such as body temperature, temporal and longitudinal changes in gastric and intestinal pH levels, peristaltic mixing and transport, gastric, biliary, and pancreatic secretions, and passive absorption of small molecules and water^95,96^. To simulate the mucus compartment, porcine stomach type III mucin secretion (Sigma-Aldrich, St. Louis, MO, USA) was added in the initial meal and delivered into the duodenum (final concentration of 3 g.L^−1^ throughout the digestive tract). In addition, two polyester pockets containing 40 mucin-alginate beads were placed in each of the four compartments of TIM-1 to provide physical surface for bacterial adhesion. In the present study, the TIM-1 model was set-up to reproduce, based on in *vivo* data, the digestive conditions of a healthy adult after ingestion of a glass of 200 mL mineral Volvic water (Table 2), contaminated with ETEC strain H10407 at a final concentration of 10^10^ CFU. Two types of in vitro digestions were performed: (i) gastric digestions where only the gastric compartment was set-up (total duration of 60 min) and (ii) gastro-intestinal digestions using the complete TIM-1 model (total duration of 300 min). During digestions, samples were regularly collected from each compartment (digestive lumen and mucin-alginate beads) to determine ETEC survival and adhesion. Gastric and ileal effluents were kept on ice and pooled on 0–10, 10–20, 20–40 and 40–60 min for gastric digestions and on 0–60, 60–120, 120–180, 180–240 and 240–300 min for gastrointestinal digestions. These effluents, as well as mucin-alginate beads, were kept at −20 °C in RNAlater™ Stabilization Solution (Invitrogen, Waltham, MA, USA) for further RNA extraction. All digestions were run in quadruplicate and control digestions were performed without any mucin secretion nor mucin beads.

**Table 2:** Parameters of the TIM-1 system when simulating digestive conditions of a healthy adult after intake of a glass of Volvic water.

TIM-1				
Parameters of in vitro digestion of a glass of water	Gastric compartment	Duodenal compartment	Jejunal compartment	Ileal compartment
pH	from 6 to 1.5 during the first 30 min	maintained at 6.4	maintained at 6.9	maintained at 7.2
Volume (mL)	200 (initial)	55	130	130
Secretions	(**i**) 130 U.min^−1^ of pepsin (**ii**) 10 U.min^−1^ of lipase (**iii**) HCl 0.5 M	**(i**) Bile solution with porcine bile extract^a^ and bile salts (27.9 mM) at 1 ml/min. After 30 min of digestion the solution is dilulted by 3. **(ii**) 20 mg/min of pancreatic juice 4 USP. (**iii**) NaHCO_3_ 0.5 M if necessary (**iv**) Mucin Type III (final concentration 3 g.L^−1^) (**v**) 3 mg of trypsin (42740 U) are directly added to the compartiment at the beginning of the experiment.	(**i**) NaHCO_3_ 0.5 M if necessary	(**i**) NaHCO_3_ 0.5 M if necessary
Half-emptying time (min) / Residence time (h)	T_1/2_ = 15 min	–	–	T_1/2_ = 150 min
Chyme mixing	water pressure	water pressure	water pressure	water pressure
Passive absorption	–	–	yes	yes
Concentration of total microbes	sterile	sterile	sterile	sterile
Oxygen level (%)	20	20	20	20
Temperature (°C)	37	37	37	37

TIM-1 model was set-up to simulate the digestive conditions of a healthy adult after ingestion of a glass of water. T_1/2_ represents the half time of gastric or ileal deliveries.

^a^45 ml of porcine bile extract at 53.2 g.L^−1^ are centrifuged (3 000 g, 15 min, 20 °C) to remove impurities. Supernatant is then diluted with 85 mL of sterile water in the final secretion solution.

### ETEC survival during gastrointestinal passage

During TIM-1 experiments, samples were taken in the initial bacterial suspension (T0 used for inoculation) and regularly collected in each compartment to determine ETEC survival by plating onto LB agar (“planktonic” bacteria). Results were expressed as percentages of initial intake and cross-compared to those obtained with an inert and non-absorbable transit marker indicating 100% survival rate for ETEC bacteria.

### Mucin-alginate beads adhesion assays during in vitro digestion

During static in vitro digestions, ETEC bacteria were allowed to adhere for 1 hour while, mucin-alginate beads were collected from the TIM-1 system at 20 and 60 min in the stomach, 120 and 240 min in the duodenum, and 180 and 300 min in both the jejunum and ileum. At the end of experiments, mucin-alginate beads were washed three times with ice-cold sterile physiological water and crushed with an ultra-turrax apparatus (IKA, Staufen, Germany). The resulting suspensions were then serially diluted and plated onto LB agar for ETEC numeration (“adhered” cells).

### Caco-2 and HT29-MTX cell culture assays

Caco-2 cells were purchased (Cell lines service, Eppelheim, Germany) and HT29-MTX cells were originated from Thecla Lesuffleur^97^. Caco-2 and HT29-MTX cells were cultivated as previously reported^98^. Both Caco-2 monoculture and Caco-2/HT29-MTX co-culture (ratio 70–30) were maintained for 18 days to reach the full differentiation stage. Cells were then infected with ETEC strain H10407 at a multiplicity of infection (MOI) of 100 for 3 h (37 °C, 5% CO_2_). At the end of the experiment, cell supernatants were collected to monitor ETEC virulence gene expression (“planktonic” bacteria). After three washes with ice-cold PBS pH 7.2 (Thermo Fisher Scientific, Waltham, MA, USA), intestinal cells were lysed with 1% Triton X-100 (Sigma-Aldrich, St. Louis, MO, USA). Serial dilutions of the lysed cells were plated onto LB agar plates to determine the number of adherent ETEC bacteria (“adhered” bacteria). Cell supernatants and lysates were also centrifuged (3000 g, 5 min, 4 °C) to discard the remaining bacterial cells. Resulting supernatants were used to measure IL-8 cytokine extra- and intracellular levels, respectively. Bacterial pellets were stored in RNA later at −20 °C for downstream analysis. All experiments were performed at least in triplicate.

### Interleukin-8 measurement by ELISA

Pro-inflammatory IL-8 cytokine concentrations were determined in the supernatants and cell lysates from the monoculture and co-culture models by ELISA according to the manufacturer’s instructions (DuoSet ELISA, human CXCL8/IL-8 ref DY208, RnD Systems, Minneapolis, MI, USA). Results were expressed as fold changes compared to control experiments without bacteria.

### RNA extractions and quality controls

Total RNA from TIM-1 samples (from digestive lumen and mucin-alginate beads) and cell culture experiments (planktonic and adhered bacteria) were extracted using the TRIzol^®^ method (Invitrogen, Waltham, MA, USA) as previously described^18^, with an additional purification step with MinElute Cleanup Kit (Qiagen, Hilden, Deutschland). Nucleic acid purity was checked and RNA was quantified using the NanoDrop ND-1000 (Thermo Fisher Scientific, Waltham, MA, USA). To remove any contaminating genomic DNA, DNAse treatment was performed as previously described^18^.

### Quantitative reverse transcription (RT-qPCR) analysis of ETEC virulence genes

RT-qPCR was performed as previously described^18^. cDNA amplification was achieved using a CFX96 apparatus (Bio-Rad, Hercules, CA, USA), using primers and conditions (40 cycles) listed in Table 3. qPCR data were analyzed using the comparative E^−ΔΔCt^ method and normalized with the reference genes *tufA* and *ihfB*. The amplification efficiency of each primer pair was determined by the generation of a standard curve based on a serial dilution of an ETEC cDNA sample. Differences in the relative expression levels of each virulence gene were calculated as follows: ΔΔCt = (Ct_target gene_ – Ct_reference gene_) _tested condition_– (Ct_target gene_ – Ct_reference gene_) _reference condition_ and data were derived from E^−ΔΔCt^.

**Table 3:** ETEC primers used in the study.

Gene	Target	Primer sequence 5’-3’	Amplicon length (pb)	References
**Genes to monitor ETEC survival by qPCR (in fecal batches)**				
*eltB*	LT toxin	F-GGCAGGCAAAAGAGAAATGG R-TCCTTCATCCTTTCAATGGCT	117	^115^
*16S*	Reference gene	F- NNNNNNNNNTCCTACGGGNGGCWGCAG R- NNNNNNNNNNTGACTACHVGGGTATCTAAKCC	464	^105^
**Genes for RT-qPCR analysis of ETEC virulence genes**				
*tufA*	Reference gene	F-GACATGGTTGATGACGAAGA R-GCTCTGGTTCCGGAATGTA	199	^116^
*ihfB*	Reference gene	F-CTGCGAGGCAGCTTCCAGTT R-GCAGCAACAGCAGCCGCTTA	419	^117^
*eltB*	LT toxin	F-GGCAGGCAAAAGAGAAATGG R-TCCTTCATCCTTTCAATGGCT	117	^115^
*leoA*	Labile enterotoxin output	F-AAACGGTGCATATCCTCGTC R-AAATGCTGCCACCGAAATAC	168	^18^
*estP*	ST toxin	F-TCTTTCCCCTCTTTTAGTCAG R- ACAGGCAGGATTACAACAAAG	165	^118^
*tolC*	TolC outer membrane protein (ST toxin secretion)	F-AAGCCGAAAAACGCAACCT R-CAGAGTCGGTAAGTGACCATC	101	^119^
*tia*	Adhesin	F-ACAGGCTTTTATGTGACCGGTAA R-GACGGAAGCGCTGGTCAGT	67	^120^
*fimH*	Minor component of Type I pili	F-GTGCCAATTCCTCTTACCGTT R-TGGAATAATCGTACCGTTGCG	164	^121^
*yghJ*	Mucinase	F-CCCTGTTAGCCGGTTGTGAT R-GGTATCGGTTCTGGCGTAGG	166	This study
*eatA*	Mucinase	F-AACGGAAGCACCGTCATTCT R-CAGAGTCAGGGAGGCGTTTT	363	This study
*rpoS*	Environmental stresses response	F-GCGCGGTAGAGAAGTTTGAC R-GGCTTATCCAGTTGCTCTGC	229	^122^
**ETEC gene quantification by RNA fluorescent in situ hybridization in batch fermentation**				
*16S*	Eubacteria 16S rRNA	1- GCTGCCTCCCGTAGGAGT 2- CGGCGTCGCTGCGTCAGG 3- MCGCARACTCATCCCCAAA	N/A	^123^
16*S*	*E. coli* 16S rRNA	1- GCAAAGGTATTAACTTTACTCCC *(Cy5 in 5’)* 2-GCAGCAACAGCAGCCGCTTA *(Helper probe)*	N/A	^103^

*F* Forward, *LT* Heat-labile enterotoxin, *R* Reverse, *ST* Heat-stable enterotoxin.

### Fecal batch experiments

Batch experiments were carried out for 24 h in 60 mL penicillin bottles containing 20 mL nutritive medium and 60 mucin-alginate beads or 60 alginate beads as a control. The nutritive medium was composed per L of: 0.5 g guar gum, 1 g pectin, 0.5 g xylan, 1 g potato starch, 1 g yeast extract, 1 g proteose peptone, and 1 g of pig gastric type III mucin (all from Sigma Aldrich, St. Louis, MO, USA), suspended into 0.1 M phosphate buffer (pH 6.8) and autoclaved before use. To examine the inter-individual variability of ETEC interactions with mucin and gut microbiota, penicillin bottles were inoculated with fecal samples collected from six healthy individuals. These donors were three males (donors 1, 2, 3) and three females (donors 4, 5, 6), ranging in age from 20 to 30 years, without any history of antibiotic, prebiotic and probiotic use 6 months prior to the study. They all gave written, informed consent to take part in the study. The Research incubation work with fecal microbiota from human origin was approved by the ethical committee of the Ghent University hospital under registration number BE670201836318. Fecal collection and fecal slurry preparation were already described in the study from De Paepe and colleagues^99^. Inoculation at a 1:5 dilution ratio of the 20% (w/v) fecal slurry resulted in a final concentration of 4% (w/v) fecal inoculum in the penicillin bottles. ETEC was pre-digested (as described above) and introduced at a final concentration of 10^8^ CFU.mL^−1^. The penicillin bottles were flushed with a gas mixture of N_2_/CO_2_ (80%/20%) during 20 cycles to obtain anaerobic conditions. The cycle was stopped at overpressure; and before starting experiments, the bottles were set at atmospheric pressure. Penicillin bottles were then incubated at 37 °C, 120 rpm on an orbital shaker KS 4000 i (IKA, Staufen, Germany). Aliquots were taken immediately after the start of the incubation (T0) and at 24 hours of fermentation from the liquid and atmospheric phases. Mucin beads were collected 24 h post-inoculation and washed twice in ice-cold physiological water before storage. All samples were immediately stored at −20 °C, except samples for flow cytometry that were fixed before storage.

### Gut microbiota metabolites analysis

SCFA production was measured using capillary gas chromatography coupled to a flame ionization detector after diethyl ether extraction, as previously described^99,100^. The gas phase composition was analyzed with a Compact gas chromatograph (Global Analyser Solutions, Breda, The Netherlands), equipped with a Molsieve 5 A precolumn and Porabond column (CH_4_, O_2_, H_2_ and N_2_) or a Rt-Q-bond pre-column and column (CO_2_). Concentrations of gases were determined with a thermal conductivity detector. The total pressure in the penicillin bottles was analyzed using a tensiometer (Greisinger, Regenstauf, Germany).

### DNA extraction

DNA extraction was performed from samples collected at T0 and T24 h during batch experiments as previously reported^99,101^. DNA quality and quantity were verified by electrophoresis on a 1.5% (w/v) agarose gel (Life technologies, Madrid, Spain) and spectrophotometer DENOVIX ds-11 (Denovix, Delaware, Wilmington).

### ETEC quantification by qPCR and RNA fluorescent in situ hybridization

qPCR was performed using StepOnePlus real-time PCR system (Applied Biosystems, Waltham, MA, USA). Reactions were conducted in a total volume of 20 μL consisting of 10 μL of 2x iTaq universal SYBR Green supermix (Bio-Rad Laboratories, Hercules, CA, USA), 2 μL of DNA template, 0.8 μL (10 µM) of each primer, and 6.4 μL nuclease-free water. Primers used to amplify cDNA are listed in Table 3. Data were analyzed using the comparative E^−ΔΔCt^ method. The amplification efficiency of the primers pair was determined by the generation of a standard curve based on serial dilution of five ETEC-infected samples. Differences in number of copy of the *eltB* gene was calculated as follows: ΔΔCt = (Ct_target gene_ – Ct_reference gene_) _sample of interest_ – (Ct_target gene_ – Ct_reference gene_) _reference sample_ and data were derived from E^−ΔΔCt^. All qPCR analyses were conducted in triplicate.

Flow cytometry samples were fixed and prepared for RNA fluorescent in situ hybridization, as previously described^102^. Cells were hybridized in 100 µL hybridization buffer for 3 h at 46 °C. The hybridization buffer consisted of 900 mmol.L^−1^ NaCl, 20 mmol.L^−1^ Tris–HCl (pH 7.2), 0.01% sodium dodecyl sulfate, 20% deionized formamide and 5 mM EDTA. The buffer also contained the two *E. coli* targeting probes at the final concentration of 2 ng.µl^−1^ and a combination of probes targeting eubacteria at the final concentration of 1 ng.µl^−1^ each (Table 3)^103^. After hybridization, samples were washed with wash buffer (900 mmol.L^−1^ NaCl, 20 mmol.L^−1^ Tris–HCl pH 7.2, 0.01% sodium dodecyl sulfate) for 15 min at 48 °C. After washing, cells were resuspended in 50 µL of PBS. Samples were diluted and stained with SYBR^®^ Green I (SG, 100x concentrate in 0.22 μm-filtered dimethyl sulfoxide, Invitrogen) and incubated for 20 min at 37 °C^104^. Samples were then analyzed immediately with an Attune NxT BRXX flow cytometer (Thermo Fisher Scientific, Waltham, MA, USA). The flow cytometer was operated with Attune™ FocusingFluid, as sheath fluid. The threshold was set on the primary emission channel of blue lasers (488 nm). After gating on the cytograms (Attune Cytometric Software), the percentage of active *E. coli* in the total bacteria population was expressed as the number of cells showing the *E. coli* probe fluorescence, out of the number of cells fluorescently labelled with the Eubacteria probes and SYBR green fluorescence. Gating strategy is presented in Supplementary Fig. 8.

### 16S Metabarcoding analysis of fecal batch microbial communities

Next-generation 16S rRNA gene amplicon sequencing of the V3-V4 region was performed by LGC Genomics (Berlin, Germany) on an Illumina MiSeq platform (Illumina), as previously described^99^. V3-V4 region of the 16S rRNA gene was amplified using modified version of the 341F (5’-CCTACGGGNGGCWGCAG-3’) and 785R (5’-GACTACHVGGGTATCTAAKCC-3’) primers derived from Klindworth and colleagues^99,105^. Luminal and mucosal samples had undergone respectively 30 and 33 amplification cycles.

All data analysis was performed in R (4.1.2). The DADA2 R package was used to process the amplicon sequence data according to the pipeline tutorial^106^. In a first quality control step, the primer sequences were removed and reads were truncated at a quality score cut-off (truncQ = 2). Besides trimming, additional filtering was performed to eliminate reads containing any ambiguous base calls or reads with high expected errors (maxEE = 2.2). After dereplication, unique reads were further denoised using the DADA error estimation algorithm and the selfConsist sample inference algorithm (with option pooling = TRUE). The obtained error rates were further inspected and after approval, the denoised reads were merged. Subsequently, the ASV table obtained after chimera removal was used for taxonomy assignment using the Naive Bayesian Classifier and the DADA2 formatted Silva v138. ASVs with a prevalence of less than 5% or corresponding to less than 50 reads in total were excluded from the analysis^107^. Rarefaction curves of samples obtained after DADA2 processing are presented in Supplementary Fig. 9.

### Statistical analysis

All statistical analysis were performed using GraphPad Prism v8.0.1, except the one conducted on the microbiota diversity composition results. Statistical data analysis on microbiota diversity was performed using R (version 4.1.2, R Core Team, 2016), using statistical packages as Phyloseq (v1.38)^108^ for ASV’s data handling, vegan v2.5.7^109^, betapart v 1.5.4 for diversity analysis of ASV’s^110^, deseq2 v1.34^111^ for significant higher/lower abundance of ASVs. The evolution of the microbial community α-diversity between conditions was followed by computing the richness (Observed ASVs) and evenness indexes (Shannon, Simpson, Inverse Simpson, Fisher) using vegan package. To highlight differences in microbial community composition between conditions, ordination and clustering techniques were applied and visualized with ggplot2 (v3.3.5)^112^. Non-metric multidimensional scaling (NMDS) was based on the relative abundance-based Bray-Curtis dissimilarity matrix^113^. The influence of the factors “ETEC infection” and the “type of beads”was determined by applying a distance-based redundancy analysis (db-RDA) using the abundance-based Bray-Curtis distance as a response variable^112,114^. db-RDA was performed both including and excluding ASV1 (attributed to *Escherichi*/*Shigella* genus by the Silva data base) from the ASV table. The significance of group separation between conditions was also assessed with a Permutational Multivariate Analysis of Variance (permANOVA) using distance matrixes^112^. Prior to this formal hypothesis testing, the assumption of similar multivariate dispersions was evaluated. In order to find statistically significant differences in ASV abundance between infected and non-infected conditions, a Wald test (corrected for multiple testing using the Benjamini and Hochberg method) was applied using the DESeq2 package. The metabolic response (measured SCFA and pH) was modelled in function of the beads and infection conditions in a db-RDA analysis.

## Supplementary information

Supplementary material

## Data Availability

All data required to evaluate the conclusions in the paper are present in the paper and/or the additional information. The sequence data have been deposited at NCBI Sequence Read Archive database with accession number PRJNA802327.

## Code availability

The code used for the 16S rRNA gene sequencing analysis (data elaboration, figures creation, and statistical analysis) is available upon request to the authors.
