# Supplementary material for: Role of mucus-bacteria interactions in Enterotoxigenic Escherichia coli (ETEC) H10407 virulence and interplay with human microbiome
Source: NPJ Biofilms Microbiomes. 2022 Oct 20;8:86. doi: 10.1038/s41522-022-00344-6 (PMC9584927; doi:10.1038/s41522-022-00344-6)
Supplement: Supplementary file 1 — Supplementary material [file 41522_2022_344_MOESM1_ESM.pdf]

Supplementary Figures

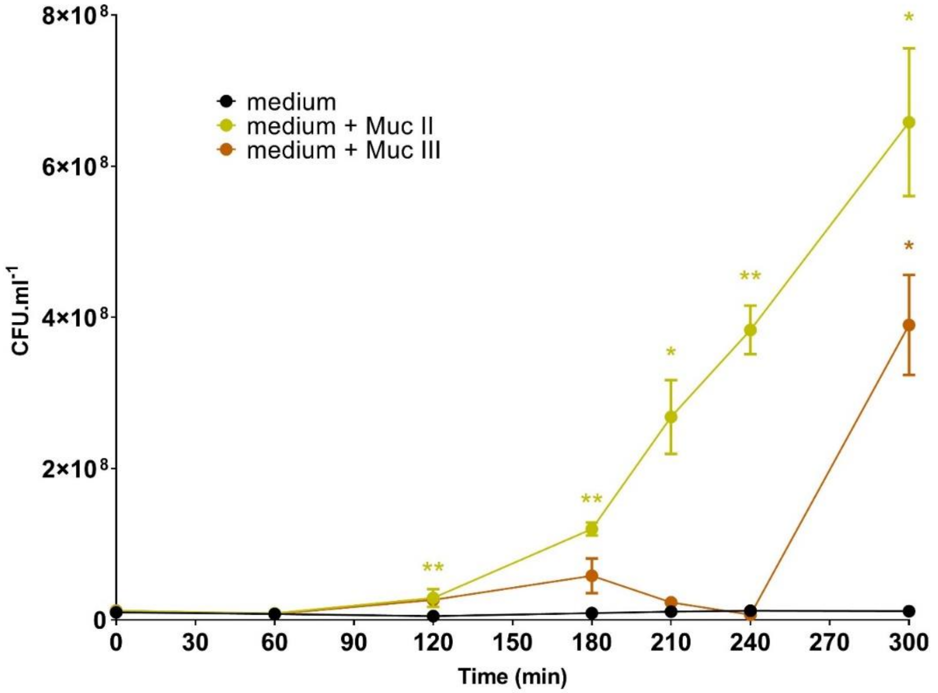

Supplementary figure 1. Impact of mucin on ETEC growth in minimal culture medium

Growth kinetics of ETEC strain H10407 (inoculation at 10^7 CFU.ml^{-1}) in M9 minimal medium supplemented with type II mucin (yellow line), type III mucin (brown line) at 3g.L^{-1} or not supplemented (black line). Samples were regularly collected and plated on LB agar. Results are expressed as CFU.mL^{-1} (mean ± SD, n=3). Statistical differences with the control condition are indicated and provided by Dunnett's multiple comparisons tests (*: $p < 0.05$; ** : $p < 0.01$) after a repeated measures ANOVA test which indicated a significant effect of mucin, time and their interaction ($p < 0.001$).

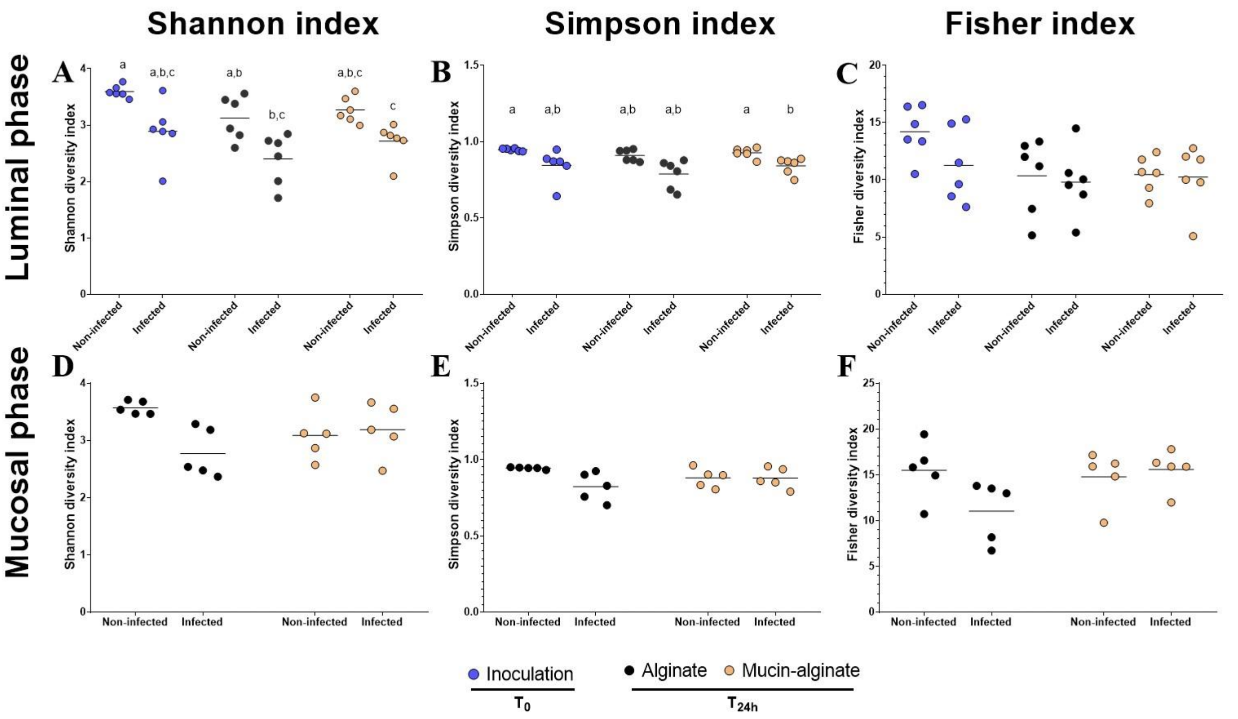

Supplementary figure 2. Impact of mucin on ETEC modulation of fecal microbial communities evenness

Batch experiments were performed using feces from 6 healthy donors, challenged or not with ETEC strain H10407, with mucin-alginate beads and alginate beads (as a control). The graphs represent the variation of the microbiota α-diversity at the ASV level in the luminal (**A-C**) and mucosal (**D-F**) compartments. The species evenness is represented by Shannon (**A, D**), Simpson (**B, E**) and Fisher (**C, F**) indexes. Blue, black and orange dots represent individual biological replicates at the beginning of the experiment (T0) or after 24 hours (T24h) in the alginate and mucin-alginate beads conditions, respectively, while black bars represent the mean. Results that are no significantly different from each other according to Tukey's multi-comparison are grouped under the same letter ($p < 0.05$).

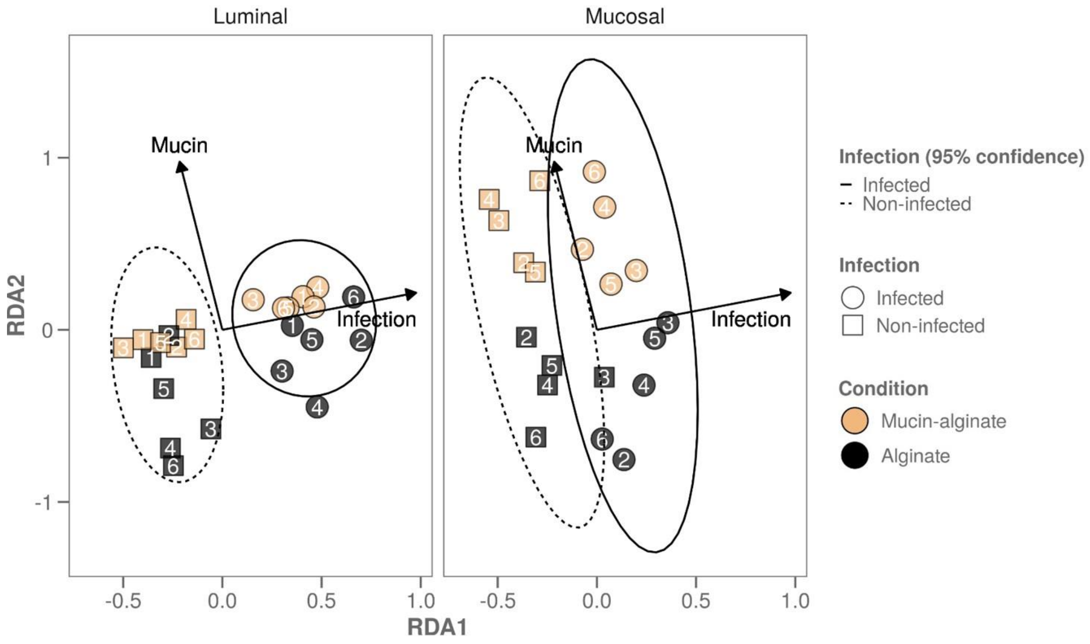

Supplementary figure 3. Distance-based redundancy analysis of the modulation of fecal microbiota β-diversity structure with the whole ASV table

Batch experiments were performed using feces from 6 healthy donors, challenged or not with ETEC strain H10407, with mucin-alginate beads or alginate beads (as a control). Distance-based redundancy analysis (db-RDA) allows the two-dimension plot visualization of the microbial community β-diversity at the ASV level, as determined by 16S rRNA gene amplicon sequencing. Compared to the db-RDA presented in Figure 7, this one includes ASV1 (attributed to the *Escherichia*/*Shigella* genus) in the relative ASV table. 'Infection' and 'mucin' were provided as sole environmental variables (binary) and plotted as vectors (arrows). Blue, black and orange dots represent individual biological replicates (donor numbers are indicated) at the beginning of the experiment (inoculation, T0) or after 24 h of fermentation (T24h) with alginate and mucin-alginate beads, respectively. Samples are represented by dot shape and square shape for the infected and non-infected conditions, respectively. The 95% confidence ellipse area is also indicated in continuous line for the infected condition and in dotted line for the non-infected condition.

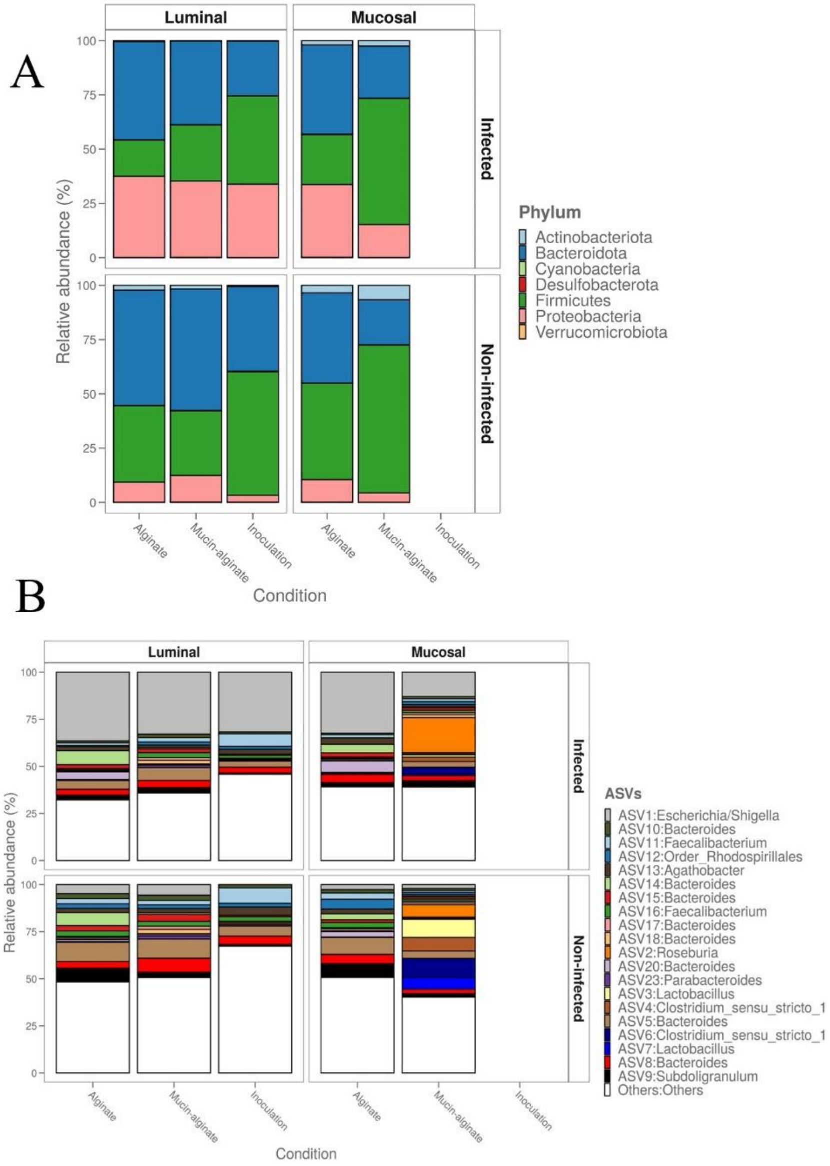

Supplementary figure 4. Impact of mucin on ETEC modulation of fecal microbial communities at the phylum and ASV levels

Batch experiments were performed using feces from 6 healthy donors, challenged or not with ETEC strain H10407, with mucin-alginate beads or alginate beads (as a control). The graph show cumulative bar plots of the relative microbial community composition at the phylum (**A**) and ASV (**B**) levels. The area graphs show the relative abundance of the 5 most abundant phyla and 20 most abundant ASV with all six different donors confounded.

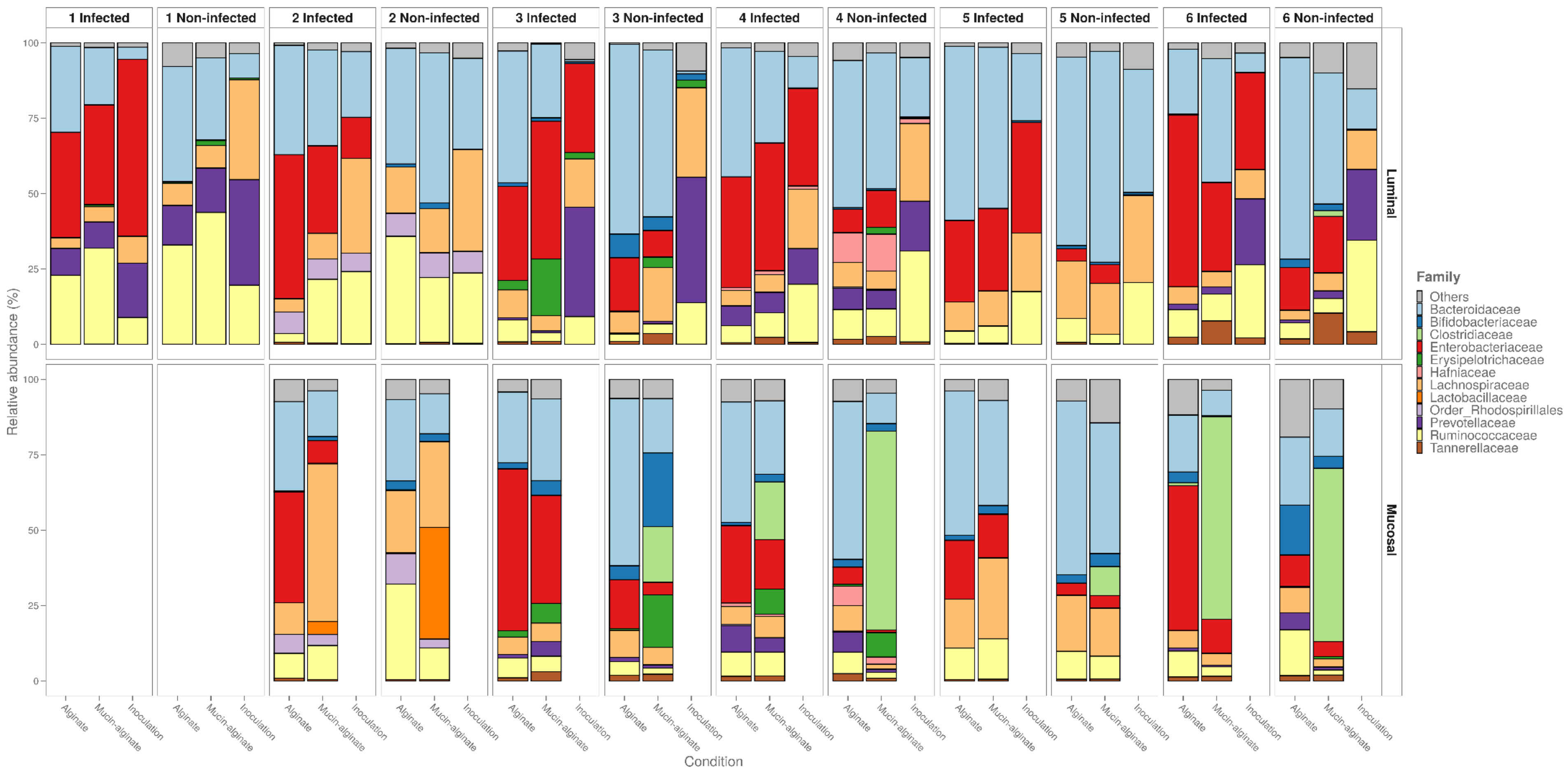

Supplementary figure 5. Donor-specific impact of mucin on ETEC modulation of fecal microbiota β-diversity at the family level

Cumulative bar plots of the relative microbial community composition in fecal batch experiments at the family level. The graphs show the relative abundance of the 12 most abundant families in the luminal and mucosal phases for the six different donors, as determined by amplicon sequencing. Mucosal phase data are missing for donor 1 due to technical problem during sampling.

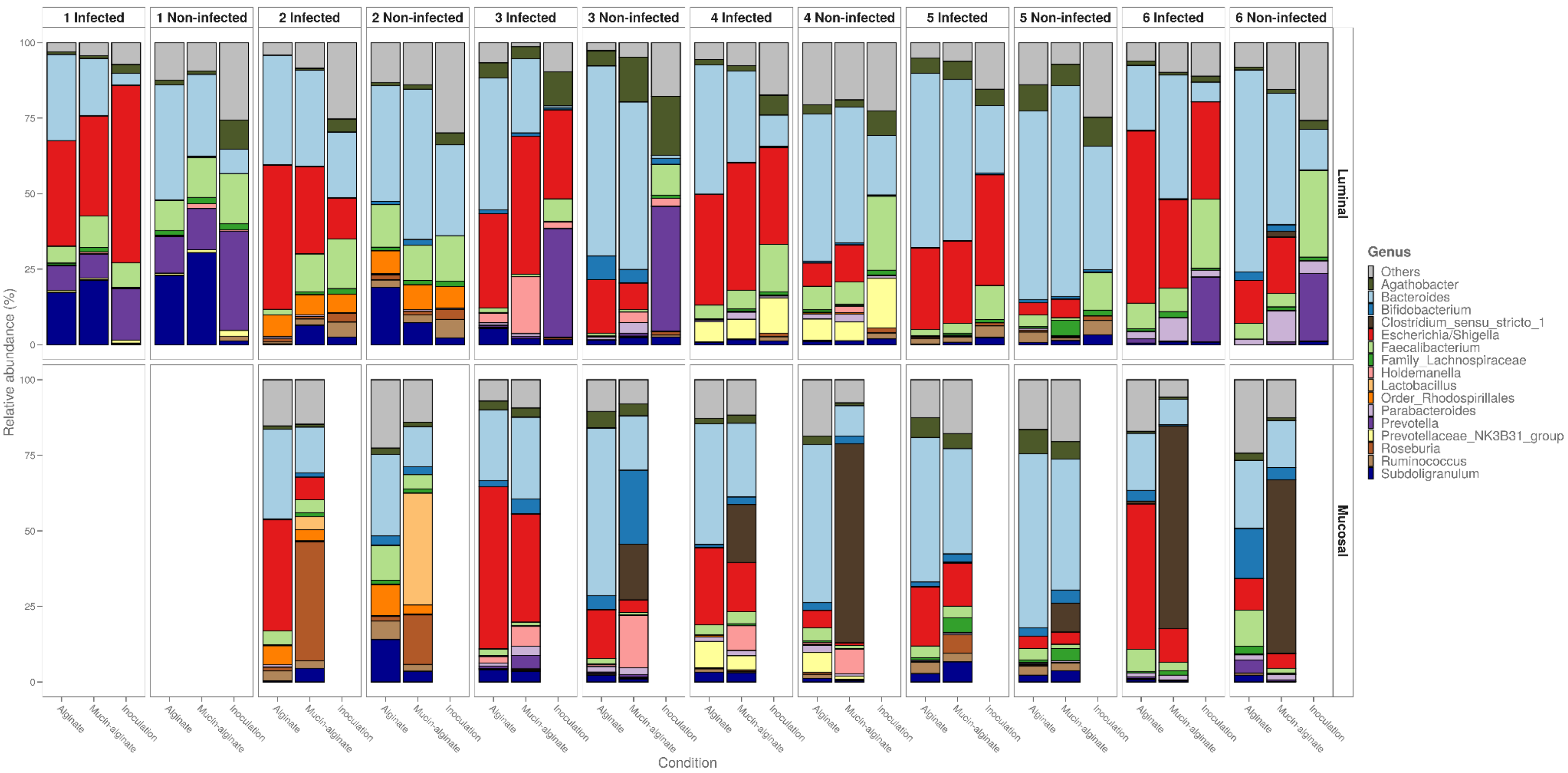

Supplementary figure 6. Donor-specific impact of mucin on ETEC modulation of fecal microbiota β-diversity at the genus level

Cumulative bar plots of the relative microbial community composition in fecal batch experiments at the family level. The graphs show the relative abundance of the 16 most abundant genera in the luminal and mucosal phases for the six different donors, as determined by amplicon sequencing. Mucosal phase data are missing for donor 1 due to technical problem during sampling.

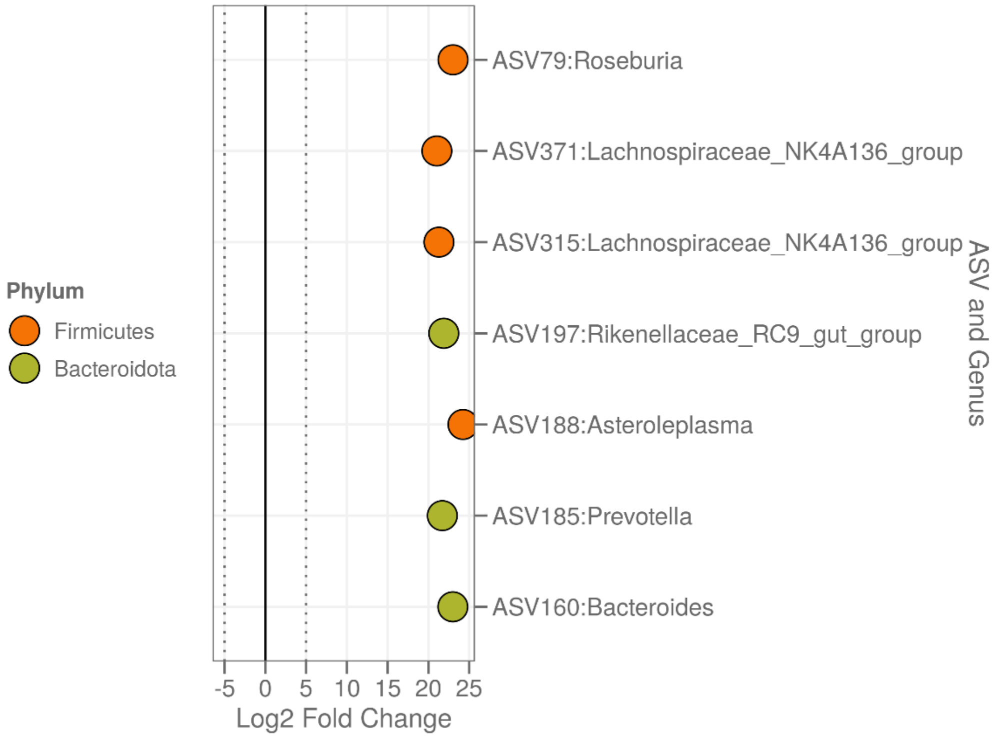

Supplementary figure 7. Log2 fold change of relative ASVs abundances significantly impacted by ETEC infection on mucin-beads microbiota in fecal batch experiments

Multivariate analysis were performed to detect which ASVs were significantly impacted by ETEC infection in the different conditions (log-transformed adjusted *p* value = 0.05). The conditions tested were the luminal phase or mucosal phase of alginate or mucin-alginate beads conditions at T24h. Only the mucosal phase of mucin beads reported some ASV with significantly affected prevalence. A positive log2 fold change indicates ASV positively correlated with ETEC infection.

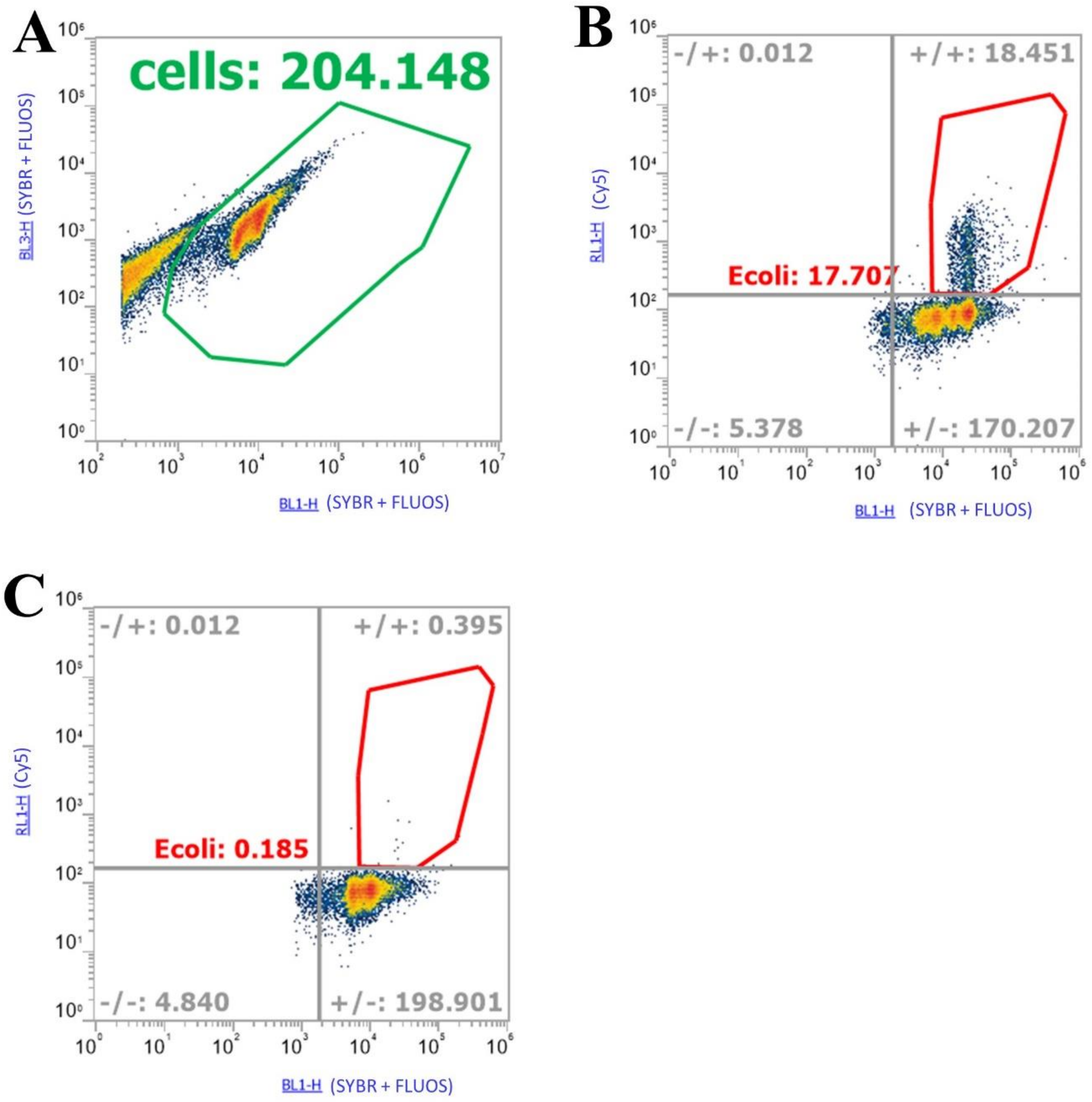

Supplementary figure 8. Gating Strategies for Flow cytometry

The samples were stained with SYBR Green and probes targeting the active E. coli and total cells population. (**A**) Total bacterial population was identified within the total count events population using the green (BL1) and red (BL3) fluorescence of the blue laser. The eubacteria probes (FLUOS) and SYBR green fluorescence overlap. (**B-C**) The active *E. coli* population was

identified within the active cells population using the red fluorescence (Cy5) of the red laser (BL3). The plot **A** and **B** represent the counting of a sample infected with ETEC, while **C** was not infected.

A

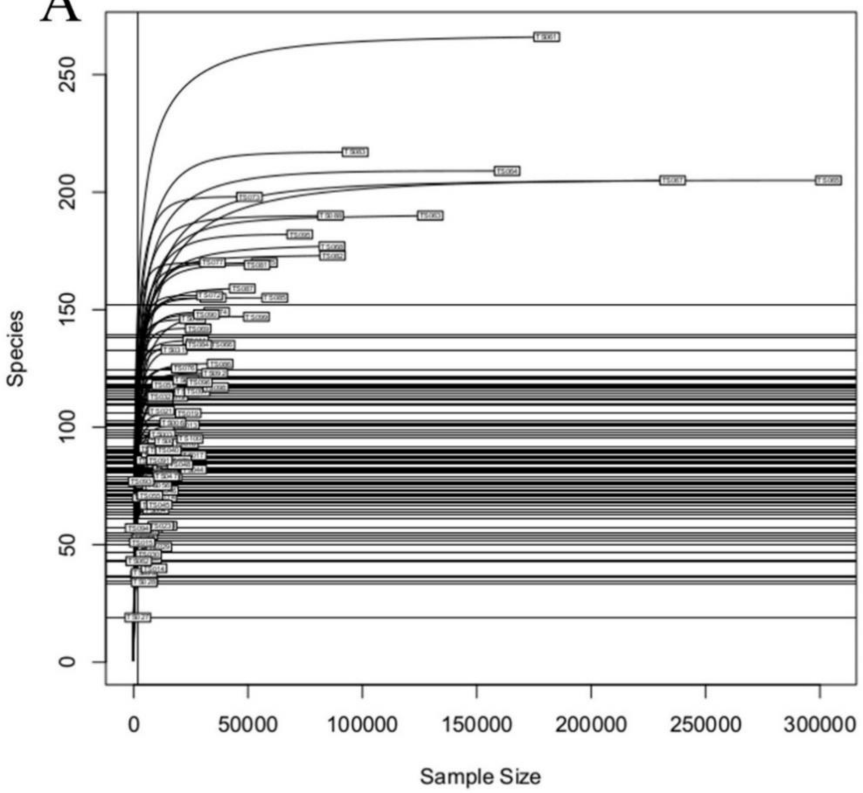
Species
0
50
100
150
200
250
0
50000
100000
150000
200000
250000
300000
Sample Size

B

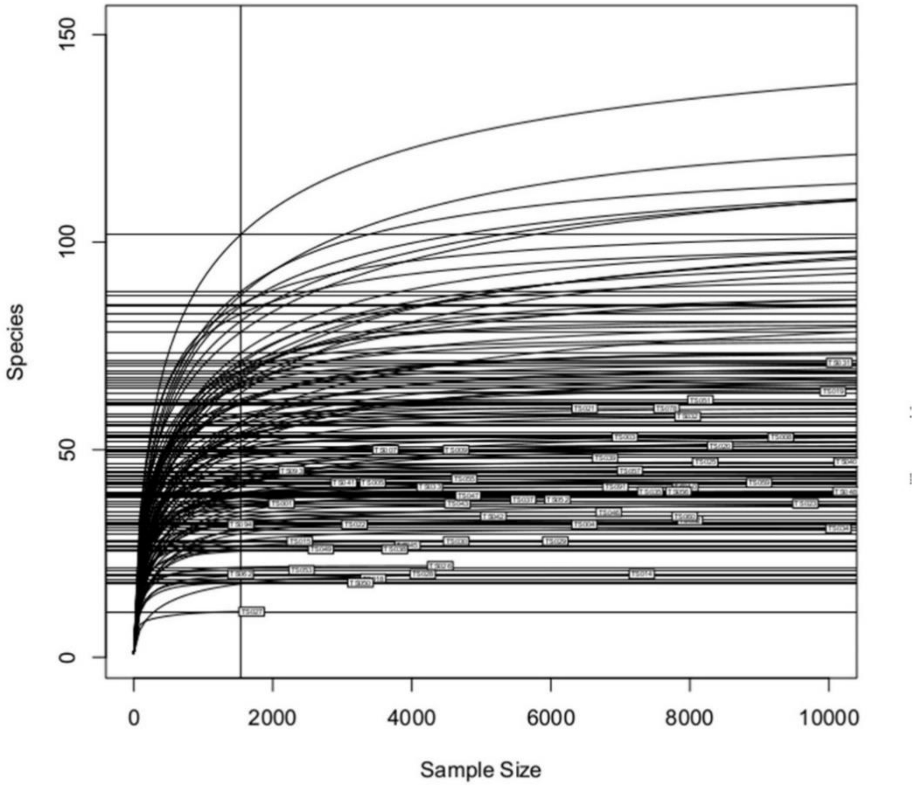
Species
0
50
100
150
0
2000
4000
6000
8000
10000
Sample Size

Supplementary figure 9. Rarefaction curves of samples after DADA2 processing. Panel **B** is a magnification of Panel **A** representing specifically the curves of the samples with less than 10 000 reads for better readability. Curves were obtained with the rarecurve function of the Stringr package.
